# CCL7 recruits cDC1 to promote antitumor immunity and facilitate checkpoint immunotherapy to non-small cell lung cancer

**DOI:** 10.1038/s41467-020-19973-6

**Published:** 2020-11-30

**Authors:** Man Zhang, Wei Yang, Peng Wang, Yu Deng, Yu-Ting Dong, Fang-Fang Liu, Rui Huang, Peng Zhang, Ya-Qi Duan, Xin-Dong Liu, Dandan Lin, Qian Chu, Bo Zhong

**Affiliations:** 1 Department of Gastrointestinal Surgery, Medical Research Institute, Zhongnan Hospital of Wuhan University, Wuhan, 430071 China; 2 Frontier Science Center for Immunology and Metabolism, Wuhan University, Wuhan, 430071 China; 3 Department of Virology, College of Life Sciences, Wuhan University, Wuhan, 430072 China; 4 Department of Thoracic Surgery, Tongji Hospital, Tongji Medical College, Huazhong University of Science and Technology, Wuhan, 430030 China; 5 Institute of Pathology, Tongji Hospital, Huazhong University of Science and Technology, Wuhan, 430030 China; 6 Department of Pathology, School of Basic Medical Science, Huazhong University of Science and Technology, Wuhan, 430030 China; 7 Department of Oncology, Tongji Hospital, Tongji Medical College, Huazhong University of Science and Technology, Wuhan, 430030 China; 8 Institute of Pathology and Southwest Cancer Center, Southwest Hospital, Third Military Medical University (Army Medical University), Chongqing, 40038 China; 9 Cancer Center, Renmin Hospital of Wuhan University, Wuhan, 430061 China

**Keywords:** Chemokines, Immunotherapy, Tumour immunology

## Abstract

The efficacy of checkpoint immunotherapy to non-small cell lung cancer (NSCLC) largely depends on the tumor microenvironment (TME). Here, we demonstrate that CCL7 facilitates anti-PD-1 therapy for the *Kras*^LSL−G12D/+^*Tp53*^fl/fl^ (KP) and the *Kras*^LSL−G12D/+^*Lkb1*^fl/fl^ (KL) NSCLC mouse models by recruiting conventional DC 1 (cDC1) into the TME to promote T cell expansion. CCL7 exhibits high expression in NSCLC tumor tissues and is positively correlated with the infiltration of cDC1 in the TME and the overall survival of NSCLC patients. CCL7 deficiency impairs the infiltration of cDC1 in the TME and the subsequent expansion of CD8^+^ and CD4^+^ T cells in bronchial draining lymph nodes and TME, thereby promoting tumor development in the KP mouse model. Administration of CCL7 into lungs alone or in combination with anti-PD-1 significantly inhibits tumor development and prolongs the survival of KP and KL mice. These findings suggest that CCL7 potentially serves as a biomarker and adjuvant for checkpoint immunotherapy of NSCLC.

## Abstract (web summary)

Only a limited proportion of patients with non-small cell lung cancer respond to anti-PD-1/PD-L1 immunotherapy. Here, the authors show that in autochthonous models of KRAS-mutated lung cancer, CCL7 promotes cDC1 infiltration into the lungs, sustaining antitumor immune responses and potentiating anti-PD1 treatment efficacy.

## Introduction

Lung cancer is the most prevalent cancer and the leading cause of cancer-related death, responsible for more than 2 million new diagnosis and 1.7 million deaths worldwide each year^1^. Approximately 85% of the diagnosed lung cancers are non-small cell lung cancer (NSCLC), with more than 50% of adenocarcinoma and 30% of squamous carcinoma. Because of the atypical symptoms, about two thirds of NSCLC patients present with advanced-stage disease at the time of diagnosis. A number of somatic mutations, oncogenic rearrangements or copy number variations in NSCLC tumors have been identified, including *EGFR* exon 19 deletions, L858R or T790M mutations, *MET* exon 14 skipping mutations, *ALK* or *ROS1* rearrangements, *MET*, *EGFR* or *HER2* copy number increases^2^. Various small molecular inhibitors and monoclonal antibodies have been developed to target these genetic alterations and significantly improve the prognosis of NSCLC patients^3–9^. Despite these advances, there are so far no specific therapeutic strategies for the NSCLC patients bearing mutations (G12C, G12V, or G12D) in *KRAS* which is the most common oncogenic driver found in 10–20% NSCLC incidences^10^. In addition, common co-mutational partners have been identified in *KRAS*-mutated NSCLC, including *TP53*, *LKB1*, and *CDKN2A*^11^. These co-mutational factors render different gene expression profiles and might determine distinct therapeutic strategies for *KRAS*-mutated NSCLCs. Mouse models which express the mutated Kras^G12D^ and simultaneously inactivate *Tp53* (*Kras*^LSL−G12D/+^*Tp53*^fl/fl^; KP) or *Lkb1* (*Kras*^LSL−G12D/+^*Lkb1*^fl/fl^; KL) have been developed and provide powerful tools for the screen and evaluation of effective therapies for *KRAS*-mutated NSCLCs^12–14^.

Recently, checkpoint immunotherapies with the immune checkpoint blockers such as anti-programmed death 1 (anti-PD-1) and anti-programmed death-ligand 1 (anti-PD-L1) antibodies have significantly improved the progression-free survival (PFS) and overall survival (OS) compared with the platinum- or docetaxel-based chemotherapies for NSCLC patients^15,16^. In addition, a more favorable prognosis of anti-PD-1/PD-L1 checkpoint immunotherapies is observed in patients with higher expression of PD-L1 in the tumor microenvironment (TME) and without *EGFR* and *ALK* mutations^17,18^, suggesting that PD-L1 expression in the TME is a critical predictive marker for checkpoint immunotherapies of NSCLC. Consistently with this notion, *LKB1* alterations are significantly associated with PD-L1 negativity and render PD-1 inhibitor resistance in *KRAS*-mutated NSCLCs^19^. However, even among the NSCLC patients with high levels of PD-L1 positivity, the objective response rate (ORR) is about 45%^17,20^, which might be due to defects in the entry or proliferation of tumor-infiltrating leukocytes or due to suppression by other molecular pathways in the TME. Therefore, administration of molecules to promote the entry, activation and expansion of tumor-infiltrating lymphocytes (TILs) would enhance the efficacy of checkpoint immunotherapies for NSCLCs.

Chemokine (C-C motif) ligand 7 (CCL7, also known as MCP-3) is a chemotactic factor and potent attractant of monocytes firstly characterized from the culture supernatants of MG-63 osteosarcoma cells^21^. CCL7 is expressed at low levels in endothelial cells, fibroblasts and mononuclear cells and upregulated by various stimuli including viruses, type I or type II interferons (IFNs)^22^. CCL7 receptors including CCR1, CCR2, and CCR3 are mainly expressed on the surface of antigen-presenting cells (APCs) such as monocytes, macrophages and dendritic cells (DCs)^23^. CCL7 deficient mice fail to efficiently eliminate various infected pathogens and exhibit impaired monocyte and neutrophil infiltration in infected tissues or organs^24,25^, indicating essential roles of CCL7 in anti-infectious immunity by recruiting immune cells to the infected microenvironment. Various studies have shown that tumor cells and stromal cells also produce high levels of CCL7, while the specific response element and signaling pathways involved are not entirely clear^26^. However, overexpression of CCL7 in tumor cells has been reported to promote tumorigenesis by facilitating tumor cell proliferation and metastasis or retain tumor growth by recruiting immune cells into tumors in exogenous mouse models^27–32^. Whether and how CCL7 is involved in primary NSCLC development in vivo are unclear.

Here, we show that high expression of CCL7 in NSCLC tumor biopsies is positively associated with the OS of NSCLC patients. CCL7 deficiency promotes NSCLC development in the KP mouse model, whereas administration of CCL7 in the lung through Lentivirus-mediated gene transfer inhibits tumorigenesis of NSCLC in the KP or KL NSCLC mouse models. Mechanistically, CCL7 promotes the chemotaxis of cDC1 into the TME which subsequently facilitates T cell expansion and antitumor immunity. Consistently, combination of CCL7 and anti-PD-1 treatment significantly inhibits tumorigenesis and prolongs the survival of KP or KL mice compared to anti-PD-1 treatment alone. These findings together suggest that CCL7 serves as an adjuvant to facilitate checkpoint immunotherapy for NSCLC by modulating the TME.

## Results

### CCL7 is highly expressed in NSCLC tumor tissues

**Fig. 1:**
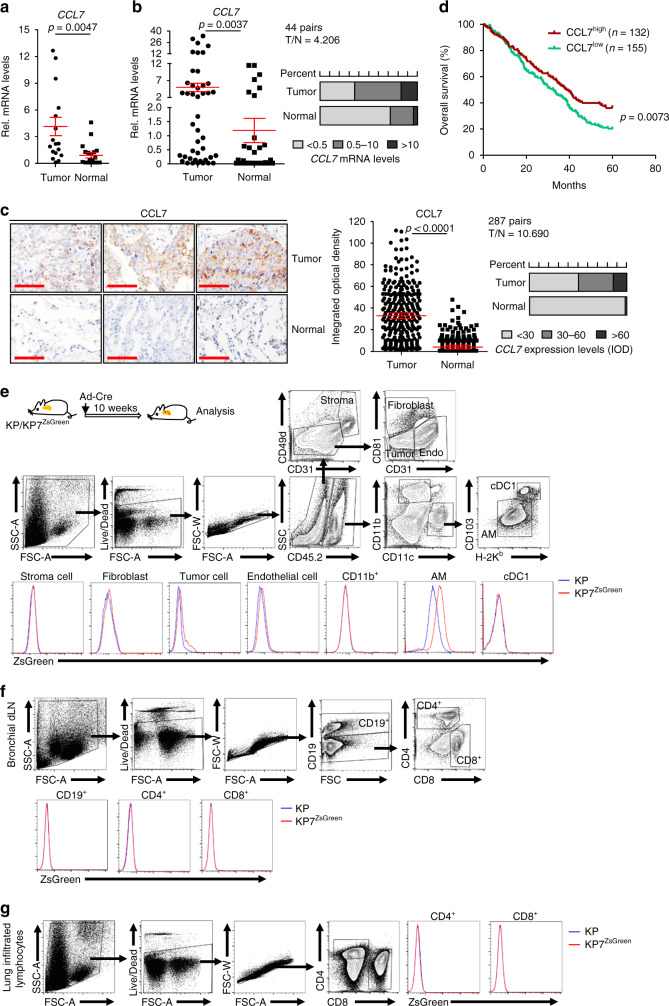
CCL7 is upregulated in NSCLC tumor tissues. **a** Quantitative real-time PCR (qRT-PCR) analysis of *CCL7* mRNA in primary tumor and adjacent normal tissues of NSCLC patients (*n* = 18). **b** qRT-PCR analysis of *CCL7* mRNA in primary tumor and adjacent normal tissues of NSCLC patients (*n* = 44). **c** Immunohistochemistry (IHC) staining (left images) and integrated optical density (IOD) analysis (right graphs) of CCL7 in tissue array of tumor and adjacent normal tissues from NSCLC patients (*n* = 287). **d** Overall survival (OS) of NSCLC patients (*n* = 287) based on the CCL7 protein levels. **e** A scheme of KP and KP7^ZsGreen^ mice intranasally injected with Ad-Cre for 10 weeks followed by flow cytometry analysis (upper scheme). Flow cytometry analysis of single-cell suspensions of lungs from KP and KP7^ZsGreen^ mice as treated in the above scheme (lower flow charts). **f** Flow cytometry analysis of immune cells in the bronchial draining lymph nodes from KP and KP7^ZsGreen^ mice treated as in **e**. **g** Flow cytometry analysis of immune cells in the lung infiltrated lymphocytes from KP and KP7^ZsGreen^ mice treated as in **e**. Two-tailed student’s *t*-test (**a**–**c**) and Log-rank analysis (**d**). T, tumor tissue; N, normal tissue; Stroma, stromal cell; Endo, endothelial cell; Tumor, tumor cell; cDC1, conventional dendritic cell 1; AM, alveolar macrophage. Graphs show mean ± SEM (**a**–**c**). Scale bars, 100 μm (**c**). Data are representative of two independent experiments (**e**–**g**). Source data are provided as a source data file.

A primary function of chemokines is to attract leukocytes mobilization^23^. We thus hypothesized that chemokines might contribute to the infiltration of immune cells into TME and thereby play critical roles in tumorigenesis and tumor development. To test this hypothesis, we initially collected 18 tumors and normal lung tissues from NSCLC patients (Cohort 1) with wedge resection or lobectomy and examined the expression of chemokines by quantitative reverse-transcription real-time PCR (qRT-PCR) assays. The results suggested that the mRNA levels of *CCL7* were significantly higher in tumor tissues than in normal tissues (Fig. 1a and Supplementary Table 1), as we have observed for *CCL28*^33^. We collected another cohort of tumors and normal lung tissues from NSCLC patients (Cohort 2) and confirmed that *CCL7* is highly expressed in tumor tissues compared to the normal lung tissues (Fig. 1b and Supplementary Tables 2 and 3), which is consistent with the data from the Gene Expression Profiling Interactive Analysis (GEPIA) (http://gepia.cancer-pku.cn/detail.php?gene=CCL7). Results from immunohistochemistry (IHC) and integrated optical density (IOD) analysis with NSCLC tissue arrays of tumor and normal lung tissues (Cohort 3) confirmed that the protein levels of CCL7 were higher in tumor tissues than in the normal lung tissues (Fig. 1c, Supplementary Data 1 and Supplementary Table 4). In addition, high CCL7 protein levels had a significantly positive correlation with the OS of NSCLC patients (Cohort 3) (Fig. 1d). These data together suggest that CCL7 is upregulated in NSCLC tumor tissues and positively correlated with the OS of NSCLC patients.

Interestingly, we found that the mRNA levels of *CCL7* were ~3.5 folds higher (*P* < 0.05) in III/IV-stage tumors than in I/II-stage tumors (Supplementary Fig. 1a). However, the protein levels of CCL7 as determined by IHC and IOD analysis were comparable between early and late-stage NSCLC tumor tissues (Supplementary Fig. 1b). We next generated the NSCLC mouse model by intranasal injection of adenovirus expressing Cre-recombinase (Ad-Cre) into *Kras*^LSL−G12D/+^*Tp53*^fl/fl^ (KP) mice and analyzed CCL7 expression in early and late-stage tumors by qRT-PCR and IHC analysis. The results showed that *Ccl7* mRNA and CCL7 protein levels were significantly higher in the lung tumors than in normal lung tissues and that *Ccl7* mRNA levels were higher in advanced tumors than in early stage tumors (Supplementary Fig. 1c, d)^34^. However, the protein levels of CCL7 were comparable in the late and early stage tumors (Supplementary Fig. 1d, e), suggesting that the expression of CCL7 is regulated at transcriptional and posttranscriptional levels.

### CCL7 is upregulated in multiple types of cells during tumorigenesis

We next generated *Ccl7*^IRES−ZsGreen^ reporter mice and found that CCL7^IRES−ZsGreen^ was undetectable in various types of cells in naive mice (Supplementary Fig. 2a–d). In contrast, CCL7^IRES−ZsGreen^ was upregulated in mouse lung fibroblasts after HSV-1 or SeV infection or IFNβ treatment (Supplementary Fig. 2e). We next obtained *Kras*^LSL−G12D/+^*Tp53*^fl/fl^*Ccl7*^IRES−ZsGreen^ (KP7^ZsGreen^) mice and induced NSCLC in these mice by intranasal injection of Ad-Cre for 10 weeks (Fig. 1e). Results from flow cytometry analysis suggested that CCL7^IRES−ZsGreen^ was expressed in CD11c^+^CD11b^−^H-2K^b−^CD103^−^ alveolar macrophages (AMs) but not in CD11c^+^CD11b^−^H-2K^b+^CD103^+^ dendritic cells (DCs), CD11b^+^CD11c^−^ cells, CD19^+^ B cells, CD4^+^ or CD8^+^ T cells (Fig. 1e–g). In addition, CCL7^IRES−ZsGreen^ was found in CD45.2^−^CD31^+^CD81^−^CD49d^−^endothelial cells, CD45.2^−^CD31^−^CD81^+^CD49d^−^fibroblasts and CD45.2^−^CD31^−^CD81^−^CD49d^−^tumor cells but not in CD45.2^−^CD31^+^CD49d^+^ stromal cells (Fig. 1e). These data together demonstrate that CCL7 is upregulated in multiple types of cells in the lung of the KP mice after tumor induction.

Consistently with previous observations that type I IFNs potently upregulate *CCL7* mRNA^22^, we found that type I or type II IFNs treatment or transfection of ISD45 substantialy upregulated the mRNA levels of *CCL7* or *Ccl7* in human A549 cells or in primary mouse lung epithelial cells, which was almost abolished by the JAK1 inhibitor (Supplementary Fig. 3a, b). Results from chromosome immunoprecipitation (ChIP) assays showed a direct binding of pSTAT1 on the human *CCL7* or mouse *Ccl7* gene promoters (Supplementary Fig. 3c, d). Importantly, treatment of JAK1 inhibitor in KP mice significantly downregulated the mRNA levels of *Ccl7* in the lungs at 8 weeks after tumor induction (Supplementary Fig. 3e), suggesting that CCL7 is upregulated in the tumor-burdened lungs in KP mouse model in a JAK-STAT-dependent manner.

### CCL7 deficiency promotes tumorigenesis in the KP mouse model

**Fig. 2:**
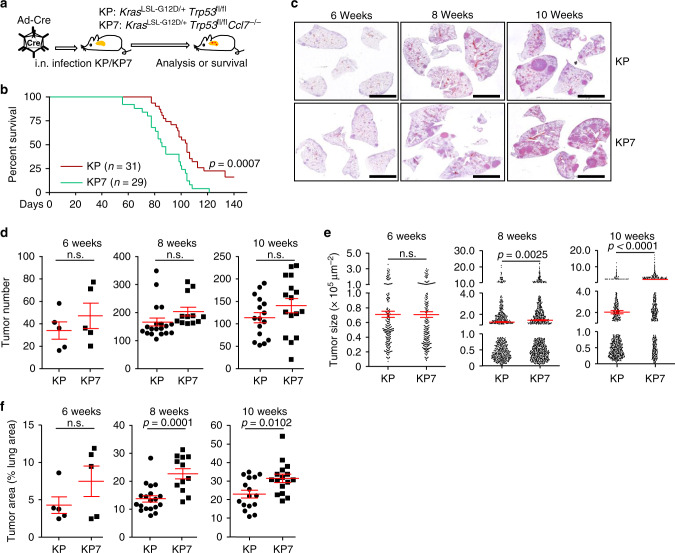
Knockout of CCL7 promotes NSCLC tumorigenesis of the KP mouse model. **a** A scheme induction of NSCLC in *Kras*^LSL−G12D/+^*Tp53*^fl/fl^ (KP) and *Kras*^LSL−G12D/+^*Tp53*^fl/fl^*Ccl7*^−/−^ (KP7) mice. KP or KP7 mice were anesthetized with sodium pentobarbital followed by intranasal injection of Ad-Cre (1.5 × 10^6^ pfu/mouse). **b** Survival of KP (*n* = 31) or KP7 (*n* = 29) mice after injection of Ad-Cre. **c** Images of HE-stained lungs of KP and KP7 mice intranasally injected with Ad-Cre for 6, 8 or 10 weeks. **d**–**f** Tumor numbers (**d**), sizes (**e**), and areas (**f**) of KP (*n* = 5, 19 or 16) and KP7 (*n* = 5, 12 or 16) mice injected with Ad-Cre for 6, 8 or 10 weeks, respectively. Log-rank analysis (**b**) or two-tailed student’s *t*-test (**d**–**f**). n.s., not significant. Graphs show mean ± SEM (**d**–**f**). Scale bars, 5 mm (**c**). Data are combined results of four independent experiments (**b**–**f**). Source data are provided as a source data file.

Since CCL7 is upregulated in NSCLC tumor tissues and positively correlated with the OS of NSCLC patients, we investigated the role of CCL7 in primary NSCLC development with the KP mouse model. The *Ccl7*^−/−^ mice were crossed with KP mice to generate *Kras*^LSL−G12D/+^*Tp53*^fl/fl^*Ccl7*^−/−^ (KP7) mice, and lung tumorigenesis was induced by intranasal injection of Ad-Cre (Fig. 2a). As shown in Fig. 2b, KP7 mice exhibited a median OS of 82 days, whereas the median OS of KP mice was significantly longer (105 days, *P* = 0.0007). In contrast, knockout of CCL28 has no effect on survival or tumor development of KP mice^33^. Histological analysis revealed that tumor numbers were comparable in the lungs of KP or KP7 mice at 6–10 weeks after tumor induction (Fig. 2c, d). In addition, the tumor sizes and areas were comparable between KP and KP7 mice at 6 weeks after induction, whereas the tumor sizes and areas were significantly larger in the lungs of KP7 mice than in those of KP mice at 8 or 10 weeks after induction (Fig. 2e, f), indicating that CCL7 deficiency does not affect the initiation of NSCLC tumors at early stage but inhibits tumor progression at late stage. However, neither ectopic expression of CCL7 in cells nor addition of CCL7 in the culture medium had any effects on cell cycle progression of A549 or HCC827 cells (Supplementary Fig. 3f). In addition, the KP and KP7 tumors displayed similar cell proliferation rate as indicated by Ki67 staining (Supplementary Fig. 3g), suggesting that CCL7 might not directly modulate tumor cell proliferation. These data collectively support a suppressive role of CCL7 in NSCLC development in KP mouse model.

### CCL7 deficiency impairs infiltration of cDC1 to tumor-burdened lung

**Fig. 3:**
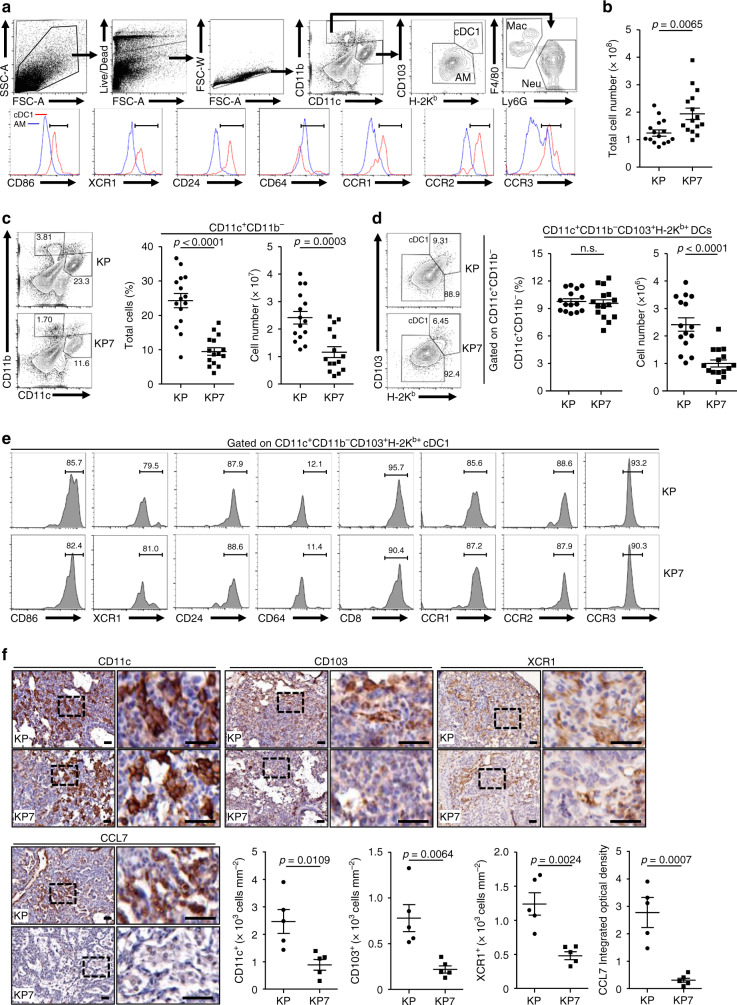
CCL7 deficiency impairs cDC1 infiltration to tumor-burdened lung. **a** Gating strategy of single-cell suspensions from lungs of KP mice that were intranasally injected with Ad-Cre (1 × 10^6^ pfu/mouse) for 10 weeks. **b** Cell numbers of tumor-burdened lungs of KP (*n* = 15) and KP7 (*n* = 15) mice intranasally injected with Ad-Cre (1 × 10^6^ pfu/mouse) for 10 weeks. **c** Flow cytometry analysis of single-cell suspensions of tumor-burdened lungs (left flow chart) and percentages and numbers of CD11c^+^CD11b^−^ cells in tumor-burdened lungs (left graphs) of KP and KP7 mice treated as in **b**. **d** Percentages and numbers of CD11c^+^CD11b^−^CD103^+^H-2K^b+^ DCs in tumor-burdened lungs of KP and KP7 mice treated as in **b**. **e** Flow cytometry analysis of CD11c^+^CD11b^−^ in **d** stained with CD103, H-2K^b^, CD86, XCR1, CD24, CD64, CCR1, CCR2, and CCR3. **f** IHC (images) and intensity or IOD (graphs) analysis of CD11c, CD103, XCR1, and CCL7 in tumor sections of KP (*n* = 5) or KP7 (*n* = 5) mice injected with Ad-Cre (1 × 10^6^) for 10 weeks. Two-tailed student’s *t*-test (**b**–**d**, **f**). n.s., not significant. Graphs show mean ± SEM (**b**–**d**, **f**). Scale bars, 50 μm. Data are combined results of four (**b**–**e**) or two (**f**) independent experiments. Source data are provided as a source data file.

Because CCL7 is a chemotactic factor for various immune cells such as monocytes and DCs, we analyzed the percentages and numbers of the myeloid cells infiltrated in the tumor-burdened lungs of KP and KP7 mice at 10 weeks after induction. Single-cell suspensions of tumor-burdened lungs from KP and KP7 mice were prepared and stained with various monocytic markers followed by flow cytometry analysis (Fig. 3a). The total cell numbers of KP7 lungs were more than those of KP lungs, which was probably due to larger tumor sizes in KP7 mice than in KP mice (Fig. 3b), whereas the total cell numbers and the percentages of CD11c^+^, CD11b^+^CD11c^−^F4/80^+^ or CD11b^+^CD11c^−^Ly6G^+^ cells in the bronchoalveolar lavage fluids (BALFs) of KP and KP7 mice were comparable (Supplementary Fig. 4a, b). Results from flow cytometry analysis suggested that the percentages and cell numbers of CD11c^+^CD11b^−^ cells were significantly reduced in lungs of KP7 mice compared to KP mice (Fig. 3c). In contrast, the numbers of CD11c^−^CD11b^+^F4/80^+^ macrophages or CD11c^−^CD11b^+^Ly6G^+^ neutrophils were comparable in lungs of KP or KP7 mice (Supplementary Fig. 4c, d). We further found that the numbers of CD11c^+^CD11b^−^CD103^+^H-2Kb^+^ DCs which expressed high levels of CD86, XCR1 and CD24 but low levels of CD64 were significantly reduced in tumor-burdened lungs of KP7 mice compared to KP mice (Fig. 3d, e). These data together suggested that CCL7 primarily attracts cDC1 in the tumor-burdened lungs in the KP mouse model. In this context, we found that CCL7 receptors CCR1, CCR2 and CCR3 were highly expressed on cDC1 compared to CD11c^+^CD11b^−^CD103^−^H-2Kb^−^alveolar macrophages or CD11c^−^CD11b^+^ cells (Fig. 3e and Supplementary Fig. 4e, f). Interestingly, we found that the numbers and percentages of CD11c^−^CD11b^−^Ly6c^+^ monocytes were comparable between the tumor-burdened lungs of KP mice and KP7 mice (Supplementary Fig. 4g, h), indicating that CCL7 is dispensable for monocytes infiltration into the lungs of KP mice after tumor induction. In this context, the CCR2 levels on monocytes were lower than those on cDC1 (Supplementary Fig. 4g). In addition, we observed that the intensities of CCL7 and CD11c^+^ cells were positively correlated in human NSCLC tumor biopsies (Supplementary Fig. 5a, b) and CD11c^hi^CCL7^hi^ patients showed significantly increased OS compared to CD11c^lo^CCL7^lo^ patients (Supplementary Fig. 5c).

Interestingly, however, we found that the percentages of CD103, H-2K^b^ were comparable on the CD11c^+^CD11b^−^ cells from the lungs of KP or KP7 mice (Fig. 3d). The cDC1 from the lungs of KP or KP7 mice also expressed comparable CD86, XCR1, CD24 or CCL7 receptors (Fig. 3e), indicating that CCL7 deficiency does not affect activation or differentiation of cDC1 or the expression of CCL7 receptors on activated cDC1. However, the numbers of cDC1 were significantly less and the intensities of CD11c, CD103 and XCR1 were significantly lower in lung tumors from KP7 mice than KP mice at 10 weeks after tumor induction (Fig. 3d, f). These data indicate that CCL7 is required for optimal infiltration of cDC1 into the tumor-burdened lungs but not for activation of the infiltrating cDC1.

### CCL7 deficiency impairs the activation of antitumor immune responses

**Fig. 4:**
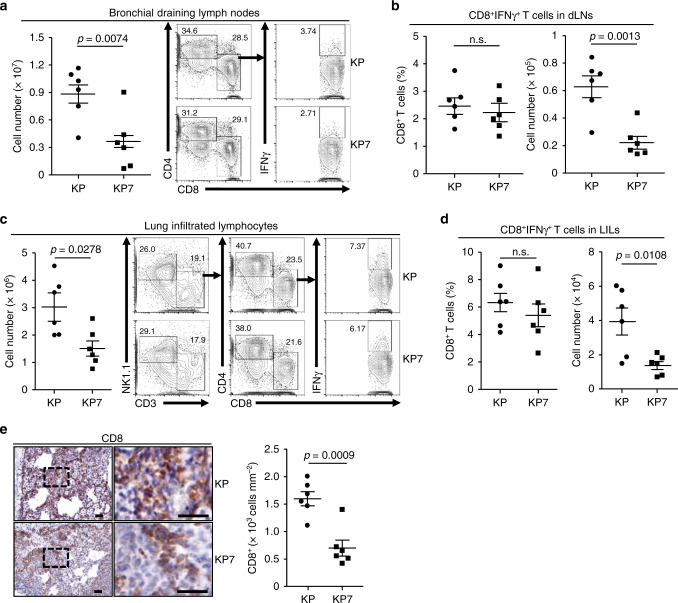
CCL7 deficiency impairs CD8^+^ T cell expansion. **a** Cell numbers and flow cytometry analysis of bronchial draining lymph nodes (dLNs) of KP (*n* = 6) and KP7 (*n* = 6) mice intranasally injected with Ad-Cre (1 × 10^6^) for 10 weeks. **b** Percentages and numbers of CD8^+^IFNγ^+^ T cells in bronchial dLNs from KP (*n* = 6) and KP7 (*n* = 6) mice treated as in **a**. **c** Lung infiltrated lymphocytes (LILs) of KP (*n* = 6) and KP7 (*n* = 6) mice injected with Ad-Cre (1 × 10^6^) for 10 weeks were isolated and calculated (left graph). LILs were stimulated with PMA and inomycin in the presence of Golgi-stop for 4 h followed by surface and intracellular staining with antibodies against NK1.1, CD3, CD4, CD8 and IFNγ and subject to flow cytometry analysis (right flow charts). **d** Percentages and numbers of CD8^+^IFNγ^+^ T cells in LILs from KP (*n* = 6) and KP7 (*n* = 6) mice treated as in **a**. **e** IHC staining (left images) and intensity or IOD analysis (right graph) of CD8 in tumor sections of KP (*n* = 6) and KP7 (*n* = 6) mice intranasally injected with Ad-Cre (1 × 10^6^) for 10 weeks. Two-tailed student’s *t*-test (**a**–**e**). n.s., not significant. Graphs show mean ±  SEM (**a**–**e**). Scale bars, 50 μm. Data are representative of two independent experiments. Source data are provided as a source data file.

cDC1 are professional APCs that uptake tumor antigens, migrate to lymphoid organs and activate tumor-specific naive T cells to become effector T cells^35^. We thus analyzed the adaptive immune responses in bronchial draining lymph nodes (dLNs). The results suggested that total cell numbers of bronchial dLNs from KP7 mice were significantly less than those from KP mice (Fig. 4a and Supplementary Fig. 5d). Although the percentages of CD4^+^ or CD8^+^ T cells in the bronchial dLNs were comparable between KP and KP7 mice at 10 weeks after tumor induction, the numbers of CD4^+^ or CD8^+^ T cells were significantly decreased in bronchial dLNs from KP7 mice compared to KP mice (Fig. 4b and Supplementary Fig. 6a). In addition, the percentages of CD8^+^IFNγ^+^, CD4^+^IFNγ^+^, CD4^+^IL-4^+^, or CD4^+^IL-17^+^ T cells in the bronchial dLNs were comparable between KP and KP7 mice, whereas the numbers of CD8^+^IFNγ^+^ T cells were significantly decreased and the numbers of CD4^+^IFNγ^+^, CD4^+^IL-4^+^, or CD4^+^IL-17^+^ T cells were also decreased in bronchial dLNs from KP7 mice compared to KP mice (Fig. 4b, and Supplementary Fig. 6b, c), suggesting that CCL7 deficiency impairs CD8^+^ T cell expansion in the bronchial dLNs after tumor induction in the KP mouse model.

The APC-activated tumor-specific effector T cells from lymphoid organs migrate into peripheral blood and traffic to tumor tissues where they recognize tumor antigen and result in tumor cell lysis^35^. We next isolated lung infiltrated lymphocytes (LILs) from the tumor-burdened lungs of KP and KP7 mice by Percoll-mediated gradient centrifugation and stained with various lymphocyte markers followed by flow cytometry analysis. The results suggested that total cell numbers of lung LILs were significantly decreased in KP7 lungs compared to KP lungs at 10 weeks after tumor induction (Fig. 4c and Supplementary Fig. 5d). The numbers of CD4^+^, CD8^+^ or CD8^+^IFNγ^+^ T cells were significantly decreased and the numbers of CD4^+^IFNγ^+^, CD4^+^IL-4^+^, or CD4^+^IL-17^+^ T cells were decreased in LILs of KP7 mice compared to KP mice (Fig. 4d, e and Supplementary Fig. 6d, e). Interestingly, consistently with a recent report^36^, the CCL7 receptors were barely expressed on CD4^+^ or CD8^+^ T cells (Supplementary Fig. 6f), indicating that decreased infiltration of CD4^+^ or CD8^+^ cells in lungs of KP7 mice is not due to a defective direct recruitment by CCL7 deficiency. We further found that the numbers and percentages of various immune cells in lungs, spleens, peripheral lymph nodes and peripheral blood were comparable between KP and KP7 mice without tumor induction (Supplementary Fig. 7). The proliferation of naive CD4^+^ or CD8^+^ T cells after plate-bound anti-CD3/CD-28 stimulation were not affected by CCL7 deficiency (Supplementary Fig. 8a). Results from bone marrow chimeric mice infected with LCMV (Armstrong) suggested that knockout of CCL7 in immune cells did not affect the activation and differentiation of CD8^+^ T cells after LCMV infection (Supplementary Fig. 8b, c), indicating that CCL7 deficiency does not directly inhibit CD8^+^ T cell activation or differentiation. In this context, the percentages of IFNγ^+^ or PD-1^+^ CD8^+^ T cells or IFNγ^+^, IL-4^+^, or IL-17^+^CD4^+^ T cells in the bronchial dLNs and LILs were comparable between KP and KP7 mice (Fig. 4b, d and Supplementary Fig. 6b, c, e, g). Collectively, these data suggest that CCL7 deficiency impairs T cell expansion and accumulation in bronchial dLNs and tumor-burdened lungs of KP mouse model.

### Depletion of CD11c^+^ or Zbtb46^+^ DCs promotes NSCLC development in KP mouse model

**Fig. 5:**
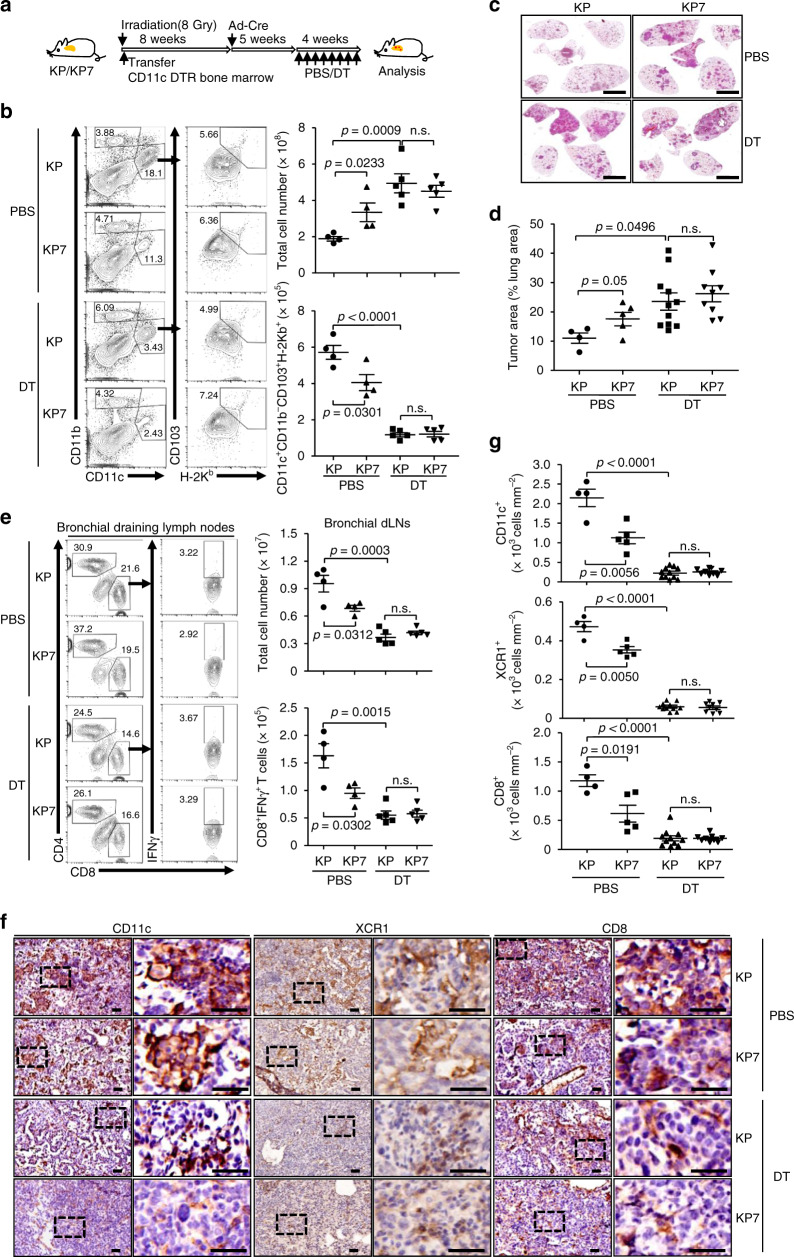
Depletion of CD11c^+^ DCs promotes tumorigenesis of KP mice. **a** A scheme of adoptive transfer of CD11c-DTR bone marrow cells and DT-mediated depletion of CD11c^+^ DC depletion in KP or KP7 mice after tumor induction. **b** Flow cytometry analysis of single-cell suspensions of tumor-burdened lungs from KP (*n* = 4 and 5 for PBS and DT, respectively) and KP7 (*n* = 4 and 5 for PBS and DT, respectively) mice treated as in **a** stained with CD11b, CD11c, CD103, and H-2K^b^ (left flow charts). The numbers of CD11c^+^CD11b^−^CD103^+^H-2K^b+^ DCs were calculated (right graphs). **c**, **d** HE-stained lungs (**c**) and tumor burden analysis of the lungs (**d**) from KP (*n* = 4 and 11 for PBS and DT, respectively) and KP7 (*n* = 5 and 9 for PBS and DT, respectively) mice treated as in **a**. **e** Flow cytometry analysis of CD3^+^ cells in bronchial dLNs of KP (*n* = 4 and 5 for PBS and DT, respectively) and KP7 (*n* = 4 and 5 for PBS and DT, respectively) mice treated as in **a** stained with CD4, CD8, and IFNγ (left flow charts). The numbers of total cells and CD8^+^IFNγ^+^ cells in dLNs were calculated (right graphs). **f**, **g** IHC (**f**) and intensities (**g**) analysis of CD11c and CD8 in tumors from KP (*n* = 4 and 11 for PBS and DT, respectively) or KP7 (*n* = 5 and 9 for PBS and DT, respectively) mice treated as in **a**. Two-tailed student’s *t*-test (**b**, **d**, **e**, **g**). n.s., not significant. Graphs show mean  ± SEM (**b**, **d**, **e**, **g**). Scale bars, 50 μm. Data are representative of two independent experiments. Source data are provided as a source data file.

Since CCL7 receptors are barely expressed on CD8^+^ T cells, we reasoned that the decreased CD8^+^ T cells in LILs were a result of CCL7 deficiency-caused insufficient cDC1 infiltration in the tumor-burdened lung of KP7 mice. To test this hypothesis, we transferred CD11c-Diphtheria Toxin Receptor (DTR) bone marrow cells into irradiated KP or KP7 mice followed by tumor induction and DT-mediated depletion of CD11c^+^ cells (Fig. 5a). As shown in Fig. 5b, the CD11c^+^ or CD11c^+^ CD103^+^H-2K^b+^ DCs in the lungs of KP or KP7 mice were efficiently depleted by treatment of DT. Depletion of CD11c^+^ DCs substantially promoted NSCLC development in both KP and KP7 mice (Fig. 5c, d). Interestingly, the tumor burden and growth were comparable in DT-treated KP and KP7 mice (Fig. 5c, d), indicating essential roles of CCL7-mediated recruitment of CD11c^+^ DCs in suppression of tumorigenesis in the KP mouse model. Consistently, CD8^+^IFNγ^+^ T cells in the bronchial dLNs and LILs and the intensities of CD8, XCR1 and CD11c stainings in tumors from KP or KP7 mice were significantly decreased after DT treatment (Fig. 5e–g and Supplementary Fig. 9a).

We next transferred Zbtb46-DTR bone marrow cells into irradiated KP or KP7 mice followed by tumor induction and DT treatment for more specific depletion of DCs (Supplementary Fig. 9b). Expectedly, treatment of DT significantly promoted tumorigenesis in KP mice (Supplementary Fig. 9c). The numbers of total cells, CD4^+^ or CD8^+^ T cells were significantly decreased in the bronchial dLNs after DT treatment (Supplementary Fig. 9d). The stainings of XCR1 and CD8 were significantly reduced in KP mice after DT treatment compared to those after PBS treatment (Supplementary Fig. 9e, f). However, the tumor growth, cell numbers in bronchial dLNs and the intensities of XCR1 and CD8 in tumor-burdened lungs were comparable between KP and KP7 mice after DT treatment (Supplementary Fig. 9c−f). These data collectively support the notion that CCL7 is essential for the recruitment of cDCs to promote T cell antitumor immune responses in the KP mouse model.

### Administration of CCL7 in the lung inhibits NSCLC in KP mouse model

We next asked whether CCL7 could serve as a therapeutic agent for NSCLC by modulating the immune responses in the TME. We made Lentiviruses express the Cre recombinase (Lenti-Cre) or the Cre recombinase and CCL7 (Lenti-Cre-CCL7) and intranasally injected into KP mice (Supplementary Fig. 10a). Administration of CCL7 significantly prolonged the survival of KP mice and inhibited tumor development at 8 weeks after tumor induction (Supplementary Fig. 10b, c). Expectedly, CCL7 significantly promoted the infiltration of cDC1 in the tumor-burdened lungs (Supplementary Fig. 10d). In addition, the numbers of CD8^+^IFNγ^+^ T cells in bronchial dLNs and LILs were significantly increased in CCL7-injected KP mice compared to the controls (Supplementary Fig. 10e). These data suggest that CCL7 promotes cDC1 and CD8^+^ T cells infiltration and expansion to elicit antitumor immunity.

**Fig. 6:**
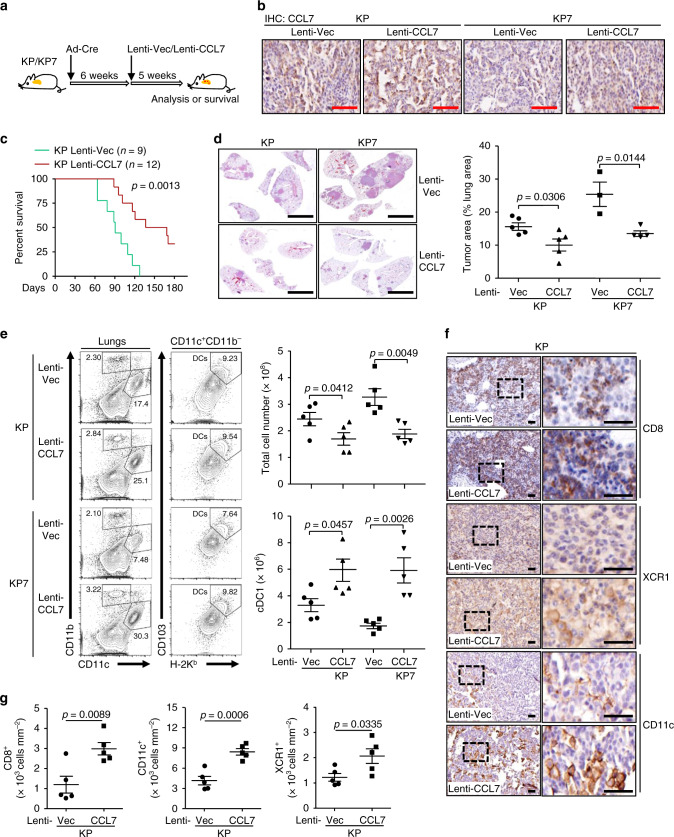
Administration of CCL7 inhibits tumorigenesis in KP mouse model. **a** A scheme of administration of CCL7 in tumor-burdened KP or KP7 mice. KP or KP7 mice were intranasally injected with Ad-Cre (1.5 × 10^6^ pfu/mouse) for 6 weeks, followed by intranasal injection of Lenti-Vec or Lenti-CCL7 for 5 weeks before analysis. **b** IHC staining of CCL7 in lung sections of KP (*n* = 5 for Vec or CCL7) and KP7 (*n* = 3 or 4 for Vec or CCL7, respectively) mice treated as in **a**. **c** Kaplan–Meier survival analysis of KP mice infected with Lenti-Vec (*n* = 9) or Lenti-CCL7 (*n* = 12) (2.0 × 10^6^ pfu/mouse). **d** HE staining (left images) and tumor burden analysis (right graph) in lungs of KP (*n* = 5 for Vec or CCL7) or KP7 (*n* = 3 or 4 for Vec or CCL7, respectively) mice treated as in **a**. **e** Flow cytometry analysis of single-cell suspensions (left flow charts) and total cell numbers of tumor-burdened lungs (upper right graph) and numbers of cDC1 in tumor-burdened lungs (lower right graph) of KP (*n* = 5 for Vec or CCL7) or KP7 (*n* = 5 for Vec or CCL7) mice treated as in **a**. **f**, **g** IHC staining (**f**) and intensity or IOD analysis (**g**) of CD8, XCR1 and CD11c in tumor sections of KP (*n* = 5 for Vec or CCL7) mice treated as in **a**. Log-rank analysis (**c**) or two-tailed student’s *t*-test (**d**, **e**, **g**). Graph shows mean ± SEM (**d**, **e**, and **g**). Scale bars, 500 μm (**b**), 5 mm (**d**) and 50 μm (**f**), respectively. Data are combined results of two independent experiments (**c**) or representatives of two independent experiments (**d**–**g**). Source data are provided as a source data file.

We next designed another model to more accurately simulate the treatment of NSCLC, as high expression of CCL7 is not associated with tumor initiation and most NSCLC patients are diagnosed at late stages. To this end, KP or KP7 mice were firstly intranasally injected with Ad-Cre and 6 weeks later these mice were further intranasally injected with Lenti-Vec or Lenti-CCL7, respectively (Fig. 6a). IHC analysis confirmed the expression of CCL7 in the lungs of Lenti-CCL7 injected KP or KP7 mice (Fig. 6b). Similarly, CCL7 injection significantly prolonged the survival and inhibited tumor development of the KP or KP7 mice compared to the respective controls (Fig. 6c–e). Consistently with these observations, the numbers of infiltrating cDC1 and the intensities of CD11c, XCR1 and CD8 staining were significantly increased in the tumor-burdened lungs of KP or KP7 mice injected with CCL7 compared to those injected with control viruses (Fig. 6f, g). In addition, the numbers of CD8^+^IFNγ^+^ T cells in the bronchial dLNs and LILs of KP or KP7 mice and the intensities of CD8 in tumors of KP mice were substantially increased after injection with CCL7 compared to the controls (Supplementary Fig. 6f, g). These data together suggest that CCL7 serves as a therapeutic agent for NSCLC in KP mouse model.

### CCL7 enhances the efficacy of anti-PD-1 checkpoint immunotherapy

**Fig. 7:**
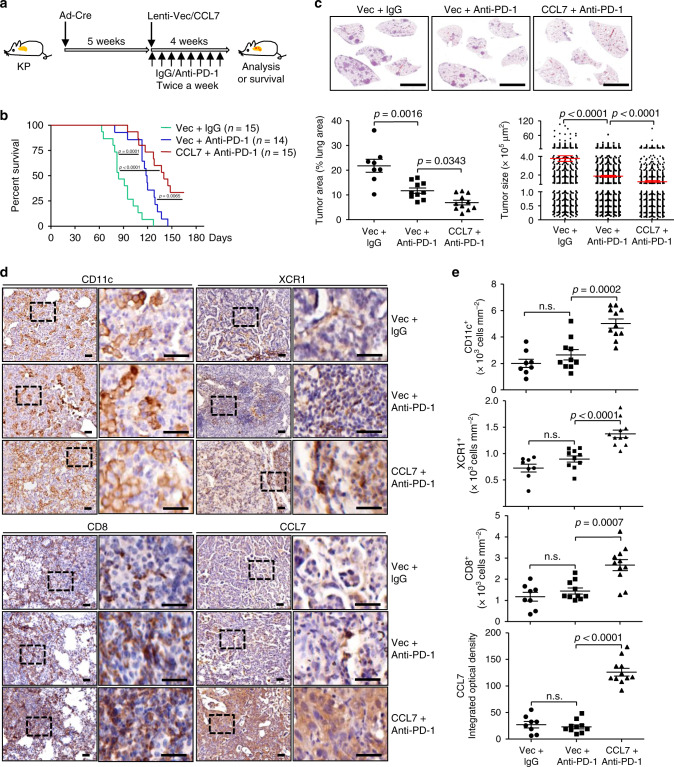
CCL7 enhances the efficacy of anti-PD-1 checkpoint immunotherapy in KP mice. **a** A scheme of combinational therapy of CCL7 and anti-PD-1. KP mice were intranasally injected with Ad-Cre (2 × 10^6^ pfu/mouse) for 5 weeks, followed by intranasal injection of Lenti-Vec or Lenti-CCL7 and intraperitoneal injection of control isotype lgG or anti-PD-1 antibody twice a week for 4 weeks. **b** Survival of KP (*n* = 15, 14, or 15 for Vec + IgG, Vec+anti-PD-1 or CCL7 + anti-PD-1, respectively) mice treated as in **a**. **c** IHC staining of sections (upper images) and tumor area (lower left graph) and size (lower right graph) analysis of tumor-burdened lungs of KP mice (*n* = 8, 10, or 11 for Vec + IgG, Vec + anti-PD-1 or CCL7 + anti-PD-1, respectively) treated as in **a**. **d**, **e** IHC staining (**d**) and intensity analysis (**e**) of CD11c, XCR1, CD8, and CCL7 in tumor sections of KP (*n* = 8, 10, or 11 for Vec + IgG, Vec + anti-PD-1 or CCL7 + anti-PD-1, respectively) mice treated as in **a**. Scale bars represent 500 μm. Log-rank analysis (**b**) or two-tailed student’s *t*-test (**c**, **e**). Scale bars, 5 mm (**c**), 50 μm (**d**), respectively. Graphs show mean ± SEM (**c**, **e**). Data are combined results of three independent experiments. Source data are provided as a source data file.

Anti-PD-1/PD-L1 checkpoint immunotherapies have been approved for NSCLC patients with PD-L1 positivity as first-line strategies and the efficacy is closely related to the infiltration of CD8^+^ T cells in the TME^37,38^. Interestingly, we observed that there was PD-L1 staining in the tumors of KP mice and that CCL7 promoted CD8^+^IFNγ^+^ T cell infiltration without affecting the expression of PD-1 on CD8^+^ T cells (Supplementary Figs. 7g and 10h). We thus speculated that anti-PD-1 treatment might promote antitumor immunity in the KP mice which could be potentiated by CCL7. To test this hypothesis, the KP mice were intranasally injected with Ad-Cre followed by Lenti-Vec or Lenti-CCL7 injections in the presence or absence of intraperitoneal injection of anti-PD-1 twice a week for 4 weeks (Fig. 7a). As shown in Fig. 7b, anti-PD-1 treatment significantly prolonged the survival of KP mice, which was further prolonged by injection of CCL7 compared to injection of the empty vector. Consistently, anti-PD-1 treatment significantly inhibited tumor development of the KP mice, which was further inhibited by combined injection of CCL7 compared to the empty vector (Fig. 7c). Consistently, combined treatment of CCL7 and anti-PD-1 significantly increased the infiltration of CD11c^+^ or XCR1^+^ DCs and CD8^+^ T cells in the lungs of KP mice compared to the anti-PD-1 treatment alone (Fig. 7d, e).

**Fig. 8:**
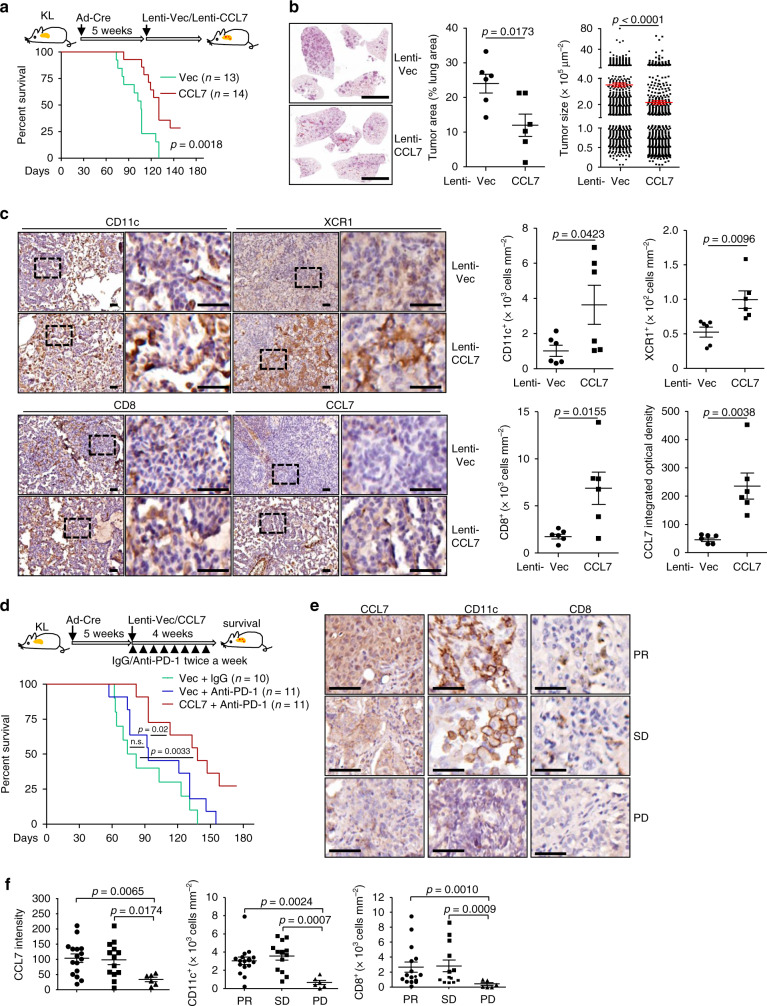
CCL7 facilitates anti-PD-1 checkpoint immunotherapy in KL mice. **a** A scheme (upper) of administration of CCL7 in tumor-burdened KL mice. KL mice were intranasally injected with Ad-Cre (1 × 10^6^ pfu/mouse) for 5 weeks, followed by intranasal injection of Lenti-Vec (*n* = 13) or Lenti-CCL7 (*n* = 14) for 5 weeks for analysis or for survival observation. Survival graph (lower) of KL mice treated as described above. **b** HE staining (left images) and tumor area and size analysis (right graphs) of KL (*n* = 6 for Vec or CCL7) mice treated as in **a**. **c** IHC staining (left images) and intensity analysis (right graphs) of KL (*n* = 6 for Vec or CCL7) mice treated as in **a**. **d** A scheme (upper) and survival analysis (lower graph) of combinational therapy of CCL7 and anti-PD-1 in KL (*n* = 10, 11, or 11 for Vec + IgG, Vec + anti-PD-1 or CCL7 + anti-PD-1, respectively) mice. KL mice were intranasally injected with Ad-Cre (2 × 10^6^ pfu/mouse) for 5 weeks, followed by intranasal injection of Lenti-Vec or Lenti-CCL7 and intraperitoneal injection of control isotype lgG or anti-PD-1 antibody twice a week for 4 weeks. **e**, **f** IHC staining (**e**) and intensity analysis (**f**) of CCL7, CD8 and CD11c in CT-guided needle biopsies from patients with advanced NSCLC received anti-PD-1 treatment. PR (*n* = 16), partial response; PD (*n* = 6), progressive disease; SD (*n* = 13), stable disease. Log-rank analysis (**a**, **d**), two-tailed student’s *t*-test (**b**, **c**, **f**). Scale bars, 5 mm (**b**) or 50 μm (**c**, **e**). Graphs show mean ± SEM (**b**, **c**, **f**). Data are combined results of two (**b**–**d**) or three (**a**) independent experiments. Source data are provided as a source data file.

We next examined whether CCL7 could promote antitumor immunity in the *Kras*^LSL−G12D/+^*Lkb1*^fl/fl^ (KL) NSCLC mouse model. KL mice were intranassaly infected with Ad-Cre and 5 weeks later the mice were infected with Lenti-Vec or Lenti-CCL7 followed by analysis or survival observation (Fig. 8a). The results showed that administration of CCL7 significantly prolonged the survival of KL mice and inhibited NSCLC development in KL mice (Fig. 8a, b). Consistently with these observations, administration of CCL7 increased the staining of CD11c, XCR1 and CD8 in tumors of KL mice (Fig. 8c). It has been reported that NSCLC patients with *KRAS* and *LKB1* mutations have poorer response to anti-PD-1 or anti-PD-L1 than those with *Kras* and *TP53* mutations^11^. In this context, we found poor but detectable expression of PD-L1 in KL tumor model (Supplementary Fig. 10h). Consistently, anti-PD-1 treatment had no obvious improvement of the survival of KL mice, whereas combination of CCL7 and anti-PD-1 significantly prolonged the survival of KL mice compared to anti-PD-1 treatment alone (Fig. 8d). Together, these data collectively suggest that CCL7 promotes cDC1-CD8^+^ T cell axis to facilitate anti-PD-1 checkpoint immunotherapy in the KP and KL NSCLC mouse models.

We next retrospectively reviewed the levels of CCL7 in CT-guided needle biopsies and the efficacy of anti-PD-1 immunotherapy in patients with advanced NSCLC (Cohort 4) who have received pembrolizumab or sintilimab treatment (Supplementary Table 5). The results suggested that the staining of CCL7, CD11c and CD8 in biopsies from patients with partial response (PR) or stable disease (SD) was significantly higher than in those from patients with progressive disease (PD) (Fig. 8e, f), indicating that CCL7 could serve as a prognostic or diagnostic marker of checkpoint immunotherapy for NSCLC.

## Discussion

The anti-PD-1/PD-L1 checkpoint immunotherapies have been approved for a broad range of cancers including NSCLC^35^. However, only a limited proportion of NSCLC patients (~20%) exhibited a durable antitumor response. The response rate to anti-PD-1/PD-L1 checkpoint immunotherapies is closely correlated to the expression of PD-L1 and the infiltrating CD8^+^ T cells in the TME^35,37,38^. Here, we show that CCL7 recruits cDC1 to promote antitumor responses and enhance the efficacy of anti-PD-1 therapy in KP and KL NSCLC mouse models, suggesting that CCL7 serve as an adjuvant for anti-PD-1 checkpoint immunotherapies. In addition, we found that the levels of CCL7 were significantly higher in patients who exhibited PR or SD to anti-PD-1 therapies than those who exhibited PD, indicating that CCL7 in addition to PD-L1 in the TME as a potential predictive marker for anti-PD-1/PD-L1 checkpoint immunotherapies of NSCLC patients.

CCL7 is originally identified in the culture supernatants of MG-63 osteosarcoma cells as an attractant for monocytes but not for neutrophils^21^. CCL7 deficiency impairs the recruitment of various immune cells such as monocytes, DCs, neutrophils, CD4^+^ and CD8^+^ T cells into the proinflammatory organs or tissues after infections^24,25,39^. We found that the infiltrated cDC1, CD4^+^, and CD8^+^ T cells in the tumor-burdened lungs were significantly decreased by CCL7 deficiency. While the cDC1 expressed high levels of CCR1/2/3, the CD4^+^ or CD8^+^ T cells barely expressed CCR1/2/3, indicating that cDC1 and CD4^+^ or CD8^+^ T cells are directly and indirectly recruited to the TME, respectively. Actually, the numbers of CD8^+^ T cells in the bronchial dLNs were significantly decreased by CCL7 deficiency, which might be due to impaired infiltration of cDC1 in the tumor-burdened lungs of KP7 mice^40^. In support of this notion, we found that depletion of CD11c^+^ or Zbtb46^+^ DCs significantly reduced CD8^+^ T cells in the bronchial dLNs. In addition, we found that the percentages of CD103, CD86, H-2K^b^, CD103, XCR1 or CD86 positivity were not affected by CCL7 deficiency, and CCL7 deficiency affected neither the homeostasis of immune cells in the lymph organs and lungs nor the activation or differentiation of CD4^+^ or CD8^+^ T cells in vitro or in vivo. These data imply that CCL7 is not involved in activation or differentiation of various immune cells, which is consistent with the observations that CD4^+^ or CD8^+^ T cells express undetectable CCR1/2/3. It should be noted that the infiltrated cDC1 in the lungs of KP7 mice was about one half of that in the lungs of KP mice, which indicates CCL7-independent alternative pathways existing for the recruitment of cDC1 to the TME. In contrast, we observed that CCL7 deficiency had no effect on the infiltration of CD11b^+^CD11c^−^F4/80^+^ macrophages or CD11b^+^CD11c^−^Ly6G^+^ neutrophils into the tumor-burdened lungs, although the CD11b^+^CD11c^−^ macrophages or neutrophils in the TME expressed certain levels of CCR1/2/3^36^. In this context, a recent report has demonstrated that NF-κB-mediated upregulation CCL2 (an agonist for CCR1/2/3) promotes tumor-associated macrophage infiltration and tumorigenesis in lung cancer^41^. Monocytes also express CCR2 and migrates into tissues by CCL2. Currently, how CCL7-CCR2 and CCL2-CCR2 signaling differentially regulates the chemotaxis of monocytes and DCs in the tumor-burdened lungs of KP model are unclear. One possible explanation for this is that monocytes express other chemokine receptors that are distinct from DCs for optimal chemotaxis in this model, which requires further investigations.

It has been shown that overexpressing CCL7 enhances the expression of CCR3 in HCT116 and HT29 cells to promote metastasis and high CCL7 expression is associated with bone metastasis of NSCLC^27,42^. However, we found that CCR1/2/3 were barely expressed on non-CD11b^+^ or non-CD11c^+^ cells in the tumor-burdened lungs of KP or KP7 mice, which is consistent with an earlier report showing that mRNAs encoding CCR1/2/3 are mostly detected in monocytic cells or neutrophils in the tumors from NSCLC patients or mice^36^. We and others have observed that advanced or late-stage NSCLC tumors expressed higher levels of *CCL7* mRNA than did the early stage NSCLC tumors^34^, which might be due to enhanced genome instability or cell death in late-stage tumors that induces a CCL7-expressing proinflammatory condition in the TME in a JAK-STAT manner^43–47^. It is thus conceivable that high *CCL7* mRNA levels are associated with poor prognosis^42^. However, results from our IHC and IOD quantification analysis suggested that CCL7 protein levels were comparable between early and late-stage tumors from NSCLC patients and KP mouse model, suggesting a posttranscriptional regulation of CCL7 protein production. In this context, the mRNA of *CD247* (encoding PD-L1) is upregulated by IFNγ and stabilized by oncogenic RAS signaling, while its translation is potentiated by MYC^48–50^. Further investigations are required to fully elucidate the mechanisms for posttranscriptional regulation of CCL7 expression in NSCLC tumors.

Therapies with the PD-1/PD-L1 blockades have significantly improved the PFS and OS compared with the chemotherapies for NSCLC patients^15,16^. Recent progresses of several phase III trials have shown that combination of anti-PD-1/PD-L1 immune checkpoint blockades plus chemo-reagents is superior to chemotherapy alone as first-line treatment for advanced NSCLC patients in regard of the OS and PFS^51^. A retrospective study shows that combination of chemotherapy and immune checkpoint inhibitors improves the treatment efficacy over immune checkpoint inhibitors alone^52^, indicating that combinational approaches would be the future standard treatment in NSCLC^53^. Our results suggested that administration of CCL7 through the intranasal pathway promoted the recruitment of cDC1 into the tumor-burdened lungs and subsequent expansion of CD8^+^ and CD4^+^ T cells in the bronchial LNs and tumor-burdened lungs. Consistently, administration of CCL7 significantly prolonged the survival of KP or KL mice and inhibited tumorigenesis in the lungs of KP or KL mice after tumor induction. In addition, combinational treatment of CCL7 and anti-PD-1 was superior to anti-PD-1 alone in regard to the survival and tumor development in lungs of KP or KL mice. Considering that that CCL7 promotes antitumor immunity in KL mouse model and patients with *LKB1* mutations have poor response to anti-PD-1/PD-L1 therapies^11^, our results indicate supplementation of CCL7 serves as a new strategy to boost or enhance the efficacy of checkpoint immunotherapies in these patients. Taken together, these findings provide evidence that CCL7 as a potential “chemo-reagent” to enhance the efficacy of immune checkpoint blockades for NSCLC patients.

## Methods

### Human NSCLC samples

Four cohorts of human NSCLC samples were collected and analyzed in this study. Cohort 1 contained 18 paired normal and tumor tissues from NSCLC patients who underwent surgery from June to August of 2013 at the Department of Thoracic Surgery, Tongji Hospital. Cohort 2 contained 44 pared tumor and normal tissues from NSCLC patients who underwent surgery from November of 2013 to March of 2014 at the Department of Thoracic Surgery, Tongji Hospital. The tumor and normal tissues (~0.2 g) were washed with PBS, immersed in TRIzol and frozen in liquid nitrogen immediately after surgery. Cohort 3 contained 287 paraffin-embedded tumor tissues collected from January of 2012 through April of 2014 at the Department of Oncology and the Department of Pathology, Tongji Hospital. Patients of cohort 3 were followed up for survival or NSCLC-related death every three months for five successive years. Cohort 4 contained 35 paraffin-embedded tumor tissues that were obtained with CT-guided needle puncture and stained as PD-L1 positive samples. Patients of cohort 4 were diagnosed with advanced NSCLC without EGFR or ALK mutations and progressed after at least one line of chemotherapy. Patients of cohort 4 were subject to pembrolizumab or sintilimab plus platinum-based chemo-reagents treatment and followed up by CT imaging to evaluate the efficacy of treatment. Patients whose tumor sizes were shrunk by more than 30% of the initial sizes were recognized as PR to the therapies. Patients whose tumor sizes were enlarged by more than 20% of the initial sizes were recognized as PD in response to the therapies. Patients whose tumor sizes were neither shrunk nor enlarged than the initial sizes were recognized as SD in response to the therapies. The clinical information of patients from the four Cohorts was included or summarized in Supplementary Tables 1–4 and Supplementary Data 1. All cases were re-reviewed by pathologists from the Department of Pathology of Tongji Hospital for the confirmation of tumor histology and tumor content. Written informed consent was obtained from all patients. This study was approved by the Institutional Research Ethic Committee of Tongji Hospital, Huazhong University of Science and Technology, and the Medical Ethic Committee of the School of Medicine, Wuhan University.

### Mice

*Kras*^LSL−G12D/+^ (#008179), *Tp53*^fl/fl^ (#008462), *Lkb1*^fl/fl^ (#014143), and *Ccl7*^−/−^ (#017638) mice were purchased from the Jackson Laboratory. *Kras*^LSL−G12D/+^, *Tp53*^fl/fl^, and *Ccl7*^−/−^ mice were crossed to obtain *Kras*^LSL−G12D/+^*Tp53*^fl/fl^ (KP) and *Kras*^LSL−G12D/+^*Tp53*^fl/fl^*Ccl7*^−/−^ (KP7) mice for maintenance and experiments. *Kras*^LSL−G12D/+^ and *Lkb1*^fl/fl^ were crossed to obtain *Kras*^LSL−G12D/+^*Lkb1*^fl/fl^ (KL) mice. The *Ccl7*^IRES−ZsGreen^ mice were generated by GemPharmatech Co., Ltd through CRISPR/Cas9-mediated genome editing. In brief, the vector encoding guide RNA (GGCACATTTCTTCAAGGCTT) was obtained by in vitro transcription and purification. The gRNAs were incubated with purified Cas9 protein and injected the fertilized eggs (one-cell stage) together with the targeting vector containing the IRES-ZsGreen cassette. The injected fertilized eggs were cultured to the two-cell stage followed by transplantation into pseudopregnant mice. The targeted genomes of F0 mice were amplified by PCR and sequenced. The genomic DNA from PCR positive F0 mice was subject to Southern blot analysis to confirm correct recombination and exclude random insertions of the targeting vector. The correct F0 mice were crossed with wild-type C57BL/6 mice to obtain the F1 *Ccl7*^IRES−ZsGreen^ mice that were crossed with the KP mice to obtain KP7^IRES−ZsGreen^ mice. The genotyping primers for the *Ccl7*^IRES−ZsGreen^ allele were listed in Supplementary Table 6. Eight-week-old male and female KP, KP7 or KL mice were intranasally injected with Ad-Cre followed by various experiments. B6.SJL (#002014) mice (8-week old) were from the Jackson Laboratory and kindly provide by Dr. Haojian Zhang (Wuhan University). CD11c-DTR mice (#004509) (8- week old) were from the Jackson Laboratory and kindly provided by Drs. Xin-Yuan Zhou and Ying Wan (Third Military Medical University). Zbtb46-DTR mice (#019506) (8-week old) were from the Jackson Laboratory and kindly provided by Drs. Cliff Yang (Sun Yat-sen University) and Xiao Shen (Zhejiang University). C57B/6 mice (8-week old) were purchased from GemPharmatech Co., Ltd (Nanjing, Jiangsu Province). All experimental groups contained male and female mice and all the mice housed in the specific pathogen-free animal facility (12 h/12 h light and dark cycle, 22 ^o^C ± 2 ^o^C) at Wuhan University. All animal experiments were performed in accordance with protocols approved by the Institutional Animal Care and Use Committee of Wuhan University.

### Quantitative real-time PCR

These experiments were performed as previously described^54,55^. Total RNA was extracted from tumor or normal tissues or cells using TRIzol reagent (Invitrogen), and the first-strand cDNA was reversed-transcribed with All-in-One cDNA Synthesis SuperMix (Biotool). Gene expression was examined with a Bio-Rad CFX Connect system (Bio-Rad CFX Manager 3.1) by a fast two-step amplification program with 2 × SYBR Green Fast qPCR Master Mix (Biotool). The value obtained for each gene was normalized to that of the gene encoding GAPDH or β-actin. Gene-specific primers are listed in Supplementary Table 6.

### Tissue microarray preparation

Tissue microarray was prepared as previously described^56^. In brief, tumor and normal tissues from NSCLC patients with wedge resection, pulmonary lobectomy or CT-guided puncture were fixed in 4% paraformaldehyde and embedded into paraffin blocks. The paraffin blocks were punched out of the selected regions based on a hematoxylin-eosin (HE) staining analysis. The punched samples were 1.5 mm in diameter and 4–6 mm in length and assembled into a new paraffin block. The tissue microarrays were sectioned (4 μm) and stained with H&E to confirm the histological results. Tumor-burdened lungs from KP or KP7 mice were fixed in 4% paraformaldehyde and embedded into paraffin blocks which were sectioned (5 μm) for subsequent analysis.

### IHC assays

The sections were deparaffinized with xylene, rehydrated in 100, 85 and 70% ethanol for 10 min, quenched for endogenous peroxidase activity in 3% hydrogen peroxide, and processed for antigen retrieval in 0.5 mM EDTA (pH8.0) buffer by heating in a microwave oven for 20 min. The sections were cooled down naturally to room temperature and stained with various antibodies diluted in PBS containing 1% BSA and incubated at room temperature for over 6 h. Immunostaining was performed using the Maixin_Bio Detection Kit peroxidase/diaminobenzidine (DAB) rabbit/mouse (Kit-9710, DAB-0031; Maixi_Bio, Fuzhou), which resulted in a brown-colored precipitate at the antigen site. Subsequently, sections were counterstained with hematoxylin (Zymed Laboratories) for 5 min and coverslipped. The information and dilution of antibodies have been listed in Table S5. Images were acquired with the Leica Aperio VERSA 8 (Aperio imagescope (v12.3.2.8013) multifunctional scanner. The intensities of DAB staining were measured and quantified with IOD or cell intensity by Image Pro Plus 6 (Media Cybernetics).

### Induction of tumorigenesis in KP or KL mouse model

Eight-to-ten-week-old KP or KP7 mice were anesthetized by intraperitoneal injection of 1% sodium pentobarbital (w/v = 1:7), followed by intranasal injection of Ad-Cre viruses (Obio Technology, Shanghai) (1~2 × 10^6^ pfu in 60 μl PBS per mouse) or Lentiviruses expressing empty vector, CCL7, Cre or Cre and CCL7 (2 × 10^6^ pfu in 60 μl PBS per mouse). The survival of mice was recorded until the end of the study. Alternatively, at the indicated time points after infection, mice were euthanized and the BALF, lungs or dLNs were removed for subsequent analysis.

### Preparation, concentration, and titration of lentiviruses

The phage-6tag vector was modified to generate Lenti-Vec, Lenti-Cre, Lenti-CCL7, or Lenti-Cre-CCL7 constructs. In brief, the gene encoding Puromycin downstream the PGK promoter was removed and the DNA encoding Cre recombinase or GFP was inserted by Pst I and Sph I. Such vectors were designated as Lenti-Cre or Lenti-Vec. The DNA encoding mouse CCL7 was inserted into the multiple clone site of the Lenti-Cre or Lenti-Vec vector with Not I and Xho I and the resulted constructs were named Lenti-Cre-CCL7 or Lenti-CCL7. The Lenti vectors were cotransfected with the package plasmids pSPAX2 and pMD2G into HEK293T cells. The medium was changed with fresh full medium (10% FBS, 1% streptomycin-penicillin and 10 μM β-mercaptoethanol) after 8 h. Forty hours later, the supernatants were harvested and filtered with a 0.45 μm filter and mixed with a Virus Precipitation Solution (5×) at 4 °C for 12 h (Cat# EMB810A-1, Excell Bio). The viruses were harvested by centrifugation at 3000 × *g* for 30 min. The supernatants were discarded and the precipitants containing Lentiviruses were re-suspended with PBS and stored at −80 °C. The resulted Lenti-Cre or Lenti-Cre-CCL7 and Lenti-Vec or Lenti-CCL7 or their serial dilutions were used to infect 3T3^loxp-RFP-stop-loxp-GFP^ cells or 3T3 cells (kindly provided by Dr. Hong-Bin Ji, Institute for Biochemistry and Cell Biology, Chinese Academy of Science, Shanghai) for forty-eight hours, respectively, and the titers was determined by flow cytometry analysis^57^.

### Isolation of mouse lung epithelial cells

Mouse primary lung epithelial cells were isolated as described previously^58^. Lungs from C57B/6 mice were perfused through cardiac lavage with PBS. Dispase solution (2 ml at 3.6 unit/ml; 17105–41; Gibco) was instilled into the lungs through a tracheal catheter. Lungs were removed from mice and incubated in the dispase solution for 1 h at room temperature. The lungs were microdissected and cell suspensions were filtered through nylon monofilament. The recovered cells were centrifuged at 1500 × *g* for 5 min and resusbended in PBS containing 1.5% FBS. The cells were incubated with anti-CD45 microbeads for 30 min at 4 °C and the CD45^+^ cells were depleted by flow-through a magnet column (Miltenyi Biotec). The resulted cells were resuspended in DMEM containing 10% FBS, 1% streptomycin-penicillin and 10 μM β-mercaptoethanol and were seeded into 48-well plates at a density of 1 × 10^5^ cells per well for overnight culture, followed by various treatments.

### Chromatin immunoprecipitation (ChIP) assays

These experiments were performed as previously described^59,60^. Cells with various stimuli were fixed with 1% formaldehyde for 15 min and washed with PBS for three times. The cells were lyzed in ChIP lysis buffer (50 mM Tris·HCl pH 8.0, 1% SDS, 5 mM EDTA) followed by sonication to generate DNA fragments of 300–500 bp. The lysates were centrifuged at 4 °C for 15 min and ChIP dilution buffer (20 mM Tris·HCl, pH 8.0, 150 mM NaCl, 2 mM EDTA, 1% Triton X-100) was added to the supernatant (4:1 volume). The resulting lysates were then incubated with protein G beads and anti-pSTAT1 (Cat9167S, CST) or control IgG at 4 °C for 4 h. DNA was eluted by ChIP elution buffer (0.1 M NaHCO3, 1% SDS, 30 μg/mL proteinase K) followed by incubation at 65 °C for overnight. The DNA was purified with a DNA purification kit (TIANGEN) and was assayed by quantitative PCR using the SFX connect system with the 2 × SYBR Green fast qPCR master mix kit (Biotool). The qPCR primer sequences of *CCL7* or *Ccl7* promoter were listed in Supplementary Table 6.

### JAK1 inhibitor treatment

KP mice intranasally injected with Ad-Cre (2 × 10^6^ pfu in 60 μl PBS per mouse) for six weeks were prepared for JAK1 inhibitor treatment. Ruxolitinib phosphate (T3043, TargetMol) was dissolved in DMSO (100 mg/ml) and diluted with PBS containing 5% (v/v) PEG300/dextrose (PEG:dex, 1:3, v/w) buffer until use. The mice were administered orally twice daily in an application dosage of 60 mg/kg bodyweight. After 2 weeks treatment of ruxolitnib, the lungs of the KP mice were separated and analyzed by qRT-RCR assay.

### Preparation of single-cell suspensions from tumor-burdened lungs

Tumor-burdened lungs from KP or KP7 mice were perfused through alveolar lavage and cardiac lavage with PBS. The lungs from one mouse were cut into small pieces (2~4 mm in diameter) and transferred into a gentleMACS C Tube with the enzyme mix containing 2.35 ml of DMEM, 100 μl of Enzyme D, 50 μl of Enzyme R, and 12.5 μl of Enzyme A from a Tumor Dissociation Kit (Miltenyi Biotech). The C Tube was tightly closed and attached onto the sleeve of the gentleMACS^TM^ Octo Dissociator (Miltenyi Biotech) with the tumor isolation program. After termination of the program, C tube was detached from the Dissociator and incubated at 37 °C for 40 min. Then repeat the tumor isolation program twice and perform a short spin up to 1500 × *g* to collect the sample at the bottom of the tube. After dissociation, the sample re-suspended was applied to MACS SmartStrainers (70 μm) to prepare single-cell suspension.

### Preparation of lung LILs

The obtained single-cell suspensions were centrifuged at 1,500 g for 5 min at room temperature, and the precipitants were re-suspended with 40% Percoll (Cat17-0891-09, GE Healthcare) in PBS (v/v). The suspension was centrifuged at 1,500 g for 20 min at room temperature and the supernatant was discarded. The precipitants containing LILs were re-suspended in 10% FBS DMEM containing PMA (50 ng/ml, P8139, Sigma), Ionomycin (500 ng/ml, I0634, Sigma), Golgi-stop (1:1000, Cat# 554724, BD Biosciences) and cultured for 4 h at 37 °C, followed by staining and flow cytometry analysis.

### Flow cytometry analysis

Flow cytometry protocol has previously described^61,62^. The single-cell suspensions of tumor-burdened lungs, bronchial dLN or the obtained LILs were re-suspended in FACS buffer (PBS, 1%BSA) and blocked with anti-mouse CD16/32 antibodies for 10 min prior to staining with the antibodies of the surface markers. For intracellular cytokine staining, cells were fixed and permealized with a fixation and permeabilization solution kit (Cat# 424401, Biolegend) followed by staining with the specific antibodies against intracellular cytokines. Antibodies used for flow cytometry analysis were listed in Table S5. Flow cytometry data were acquired on a FACSCelesta flow cytometer (BD Biosciences, BD FACSDiVa Software v8.0.1.1) and analyzed with Flowjo 10.6.2 software (TreeStar). The staining antibodies were listed in Supplementary Table 7.

### Bone marrow transfer and LCMV infection

C57BL/6 mice (8-week old) were irradiated (8 Gy, 4 Gy for twice) followed by injection of mixed bone marrow cells from CD45.1^+^ (wild-type) mice (1 × 10^6^) and CD45.2^+^ (*Ccl7*^−/−^) mice (1 × 10^6^) through tail vein. Eight weeks later, the mice were intraperitoneally injected with LCMV (2 × 10^5^ pfu per mouse) (Armstrong) which was kindly provided by Dr. Xin-Yuan Zhou (Third Military Medical University). The mice were sacrificed one week after infection and the spleenocytes were left unstimulated or stimulated with PMA and ionomycin plus Golgi stop followed by surface and intracellular staining with GP31-41 tetramer, CD45.1, CD45.2, and IFNγ and flow cytometry analysis.

### Diphtheria toxin-mediated depletion of CD11c^+^ or Zbtb46^+^ DCs

Bone marrow cells were isolated from the femur of CD11c-DTR or Zbtb46-DTR donor mice. Ten-week-old recipient KP or KP7 mice were irradiated with 8 Gy (4 Gy for twice) by small animal X-ray irradiater (RS2000Pro, Rad Source) and immediately injected the isolated CD11c-DTR or Zbtb46-DTR bone marrow cells through the tail vein (10^6^ cells per mouse). Eight weeks later, the recipient KP or KP7 mice were intranasally infected with Ad-Cre (2 × 10^6^ pfu per mouse). At the fifth week after tumor induction, the recipient KP or KP7 mice were injected intraperitoneally with DT (4 ng/g body weight, D0564, Sigma) or PBS every three days for 4 weeks. DC depletion efficiency in the recipient KP or KP7 mice were examined by flow cytometry and IHC analysis.

### Combinational treatment of CCL7 and anti-PD-1

Eight-week-old KP mice were infected intranasally with Ad-Cre (2 × 10^6^ pfu in 60 μl PBS per mouse). At fifth week after tumor induction, mice were intranasally injected with Lenti-GFP or Lenti-GFP-CCL7 (2 × 10^6^ pfu in 60 μl PBS per mouse). These mice were either intraperitoneally injected with control lgG (BE0091, BioXcell) or anti-PD-1 (J43BE0033-2, BioXcell) (0.2 mg in 200 μl PBS per mouse each time) twice a week until death or for 4 weeks for histological analysis.

### Hematoxylin-eosin staining analysis

Lungs from mice were fixed in 4% paraformaldehyde and embedded into paraffin blocks as previously described^63^. The paraffin blocks were sectioned (5 μm) for H&E staining (Beyotime Biotech) followed by coverslipped. Images were acquired using a Aperio VERSA 8 (Leica) multifunctional scanner.

### Statistical analysis

Differences between experimental and control groups were tested using Student’s *t*-test. The number of repeats for each experiment is also indicated in the respective figure legends. N in the figure legends indicates the number of mice or replicates in the experiments. *P* values < 0.05 were considered statistically significant. For animal survival analysis, the Kaplan–Meier method was adopted to generate graphs, and the survival curves were analyzed with log-rank analysis. Prism 6 was used to generate graphs and perform statistical analysis.

### Reporting summary

Further information on research design is available in the Nature Research Reporting Summary linked to this article.

## Supplementary information

Supplementary Information

Descriptions of Additional Supplementary Files

Supplementary Data 1

Reporting Summary

## Data Availability

The source data underlying Figs. 1a–c, 2b−f, 3b−d, f, 4, 5b, d–g, 6c–g, 7b, c, e and 8 and Supplementary Figs. 1, 2a, 3, 4a, c, d, h, 6a, c–g, 7, 8c, 9 and 10 are provided as a Source Data file. All the other data supporting the findings of this study are available within the article and its supplementary information files and from the corresponding author upon reasonable request. A reporting summary for this article is available as a Supplementary Information file. Source data are provided with this paper.

## Source data

Source Data
