# Supplementary material for: CCL7 recruits cDC1 to promote antitumor immunity and facilitate checkpoint immunotherapy to non-small cell lung cancer
Source: Nat Commun. 2020 Nov 30;11:6119. doi: 10.1038/s41467-020-19973-6 (PMC7704643; doi:10.1038/s41467-020-19973-6)
Supplement: Supplementary file 1 — Supplementary Information [file 41467_2020_19973_MOESM1_ESM.pdf]

Supplementary Figure 1

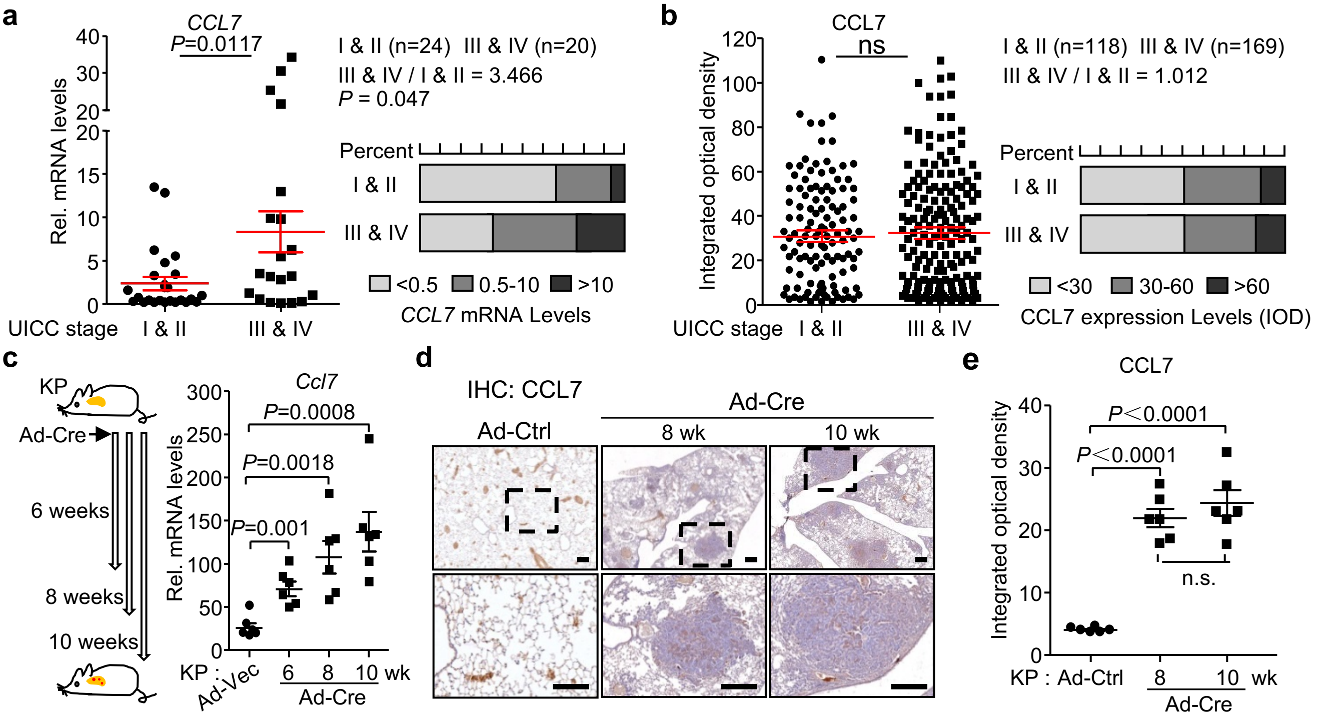

Supplementary Figure 1 CCL7 is upregulated in NSCLC tumor tissues.
(**a**) qRT-PCR analysis of *CCL7* mRNA in tumor tissues of Union for International Cancer Control (UICC) stage I-II and stage III-IV NSCLC patients (n=44).
(**b**) IHC and IOD analysis of CCL7 protein level in tumor tissues of UICC stage I-II (n=118) and stage III-IV (n=169) NSCLC patients.
(**c**) A scheme of NSCLC induction in KP mice (left) and qRT-PCR analysis of *Ccl7* mRNA levels in tumor-burdened lungs of KP mice infected with Ad-Ctrl (n=6) or Ad-Cre (n=6 for 6, 8, or 10 weeks) for 6, 8 and 10 weeks (right).
(**d, e**) IHC staining (**d**) and IDO analysis (**e**) of CCL7 expression in tumor-burdened lungs of KP mice infected with Ad-Ctrl (n=6) or Ad-Cre (n=6 for 8 or 10 weeks) for 8 and 10 weeks.
Two-tailed student's *t*-test (**a-c, e**). n.s.: not significant. Scale bars, 800 μm. Graphs show mean ± SEM (**a-c, e**). Data are representatives of two independent experiments (**c-e**). Source data are provided as a source data file.

Supplementary Figure 2

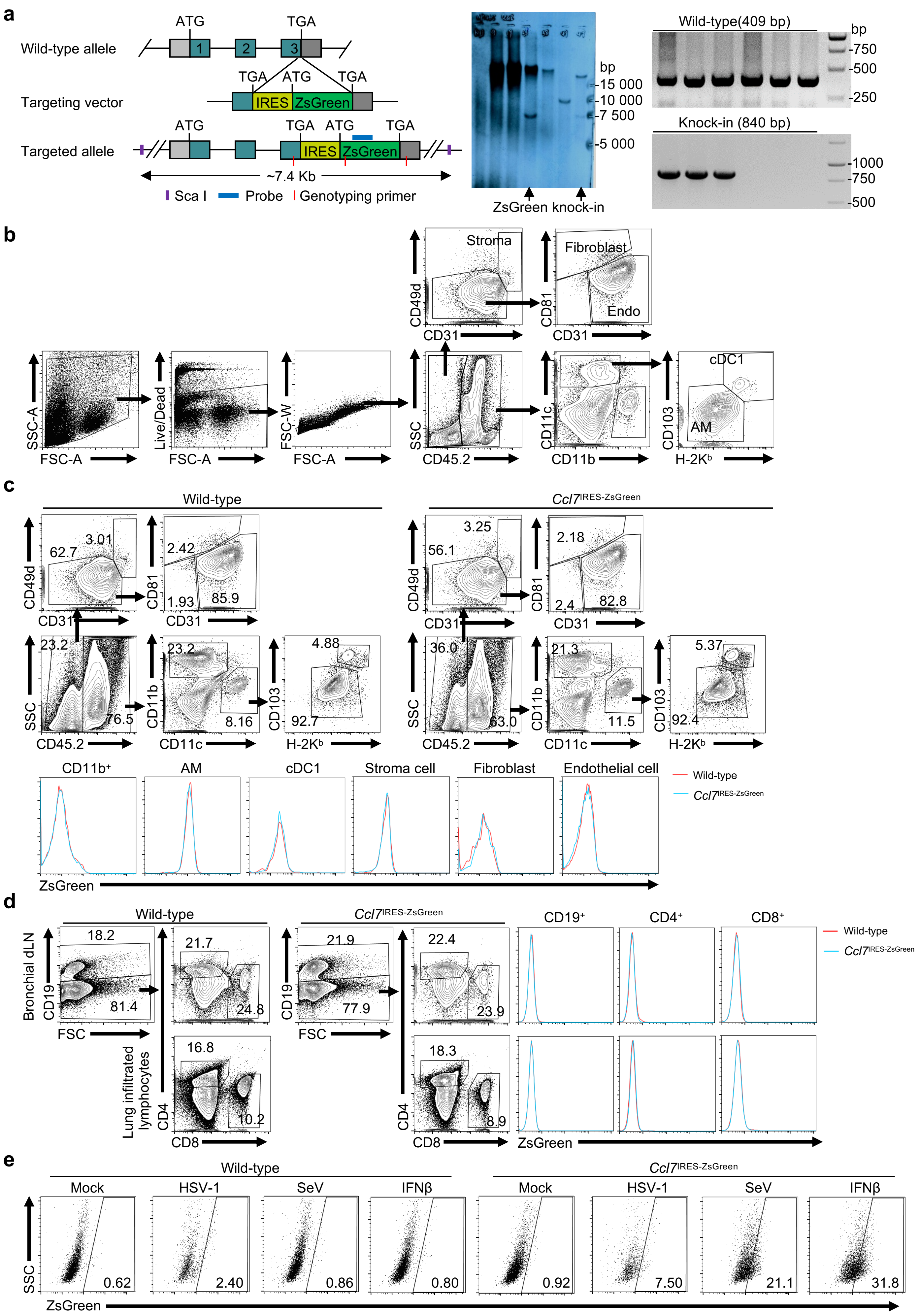

Supplementary Figure 2 Generation and analysis of *Ccl7*$^{\text{IRES-ZsGeen}}$ mice.

(**a**) A scheme of generation of *Ccl7*$^{\text{IRES-ZsGreen}}$ mice (left). Southern blot analysis of F0 *Ccl7*$^{\text{IRES-ZsGreen}}$ mice (middle). Genotyping of F1 *Ccl7*$^{\text{IRES-ZsGreen}}$ mice by PCR analysis (right).

(**b**) Gating strategy for analysis of naive wild-type and *Ccl7*$^{\text{IRES-ZsGreen}}$ mice.

(**c**) Flow cytometry analysis of single cell suspension of lungs from wild-type and *Ccl7*$^{\text{IRES-ZsGreen}}$ mice.

(**d**) Flow cytometry analysis of immune cells in the bronchial draining lymph nodes from wild-type and *Ccl7*$^{\text{IRES-ZsGreen}}$ mice.

(**e**) Flow cytometry analysis of wild-type and *Ccl7*$^{\text{IRES-ZsGreen}}$ mouse lung fibroblasts that were infected with HSV-1 or SeV for 12 h or treated with IFNβ for 6 h.

Data are representatives of two independent experiments. Source data are provided as a source data file.

Supplementary Figure 3

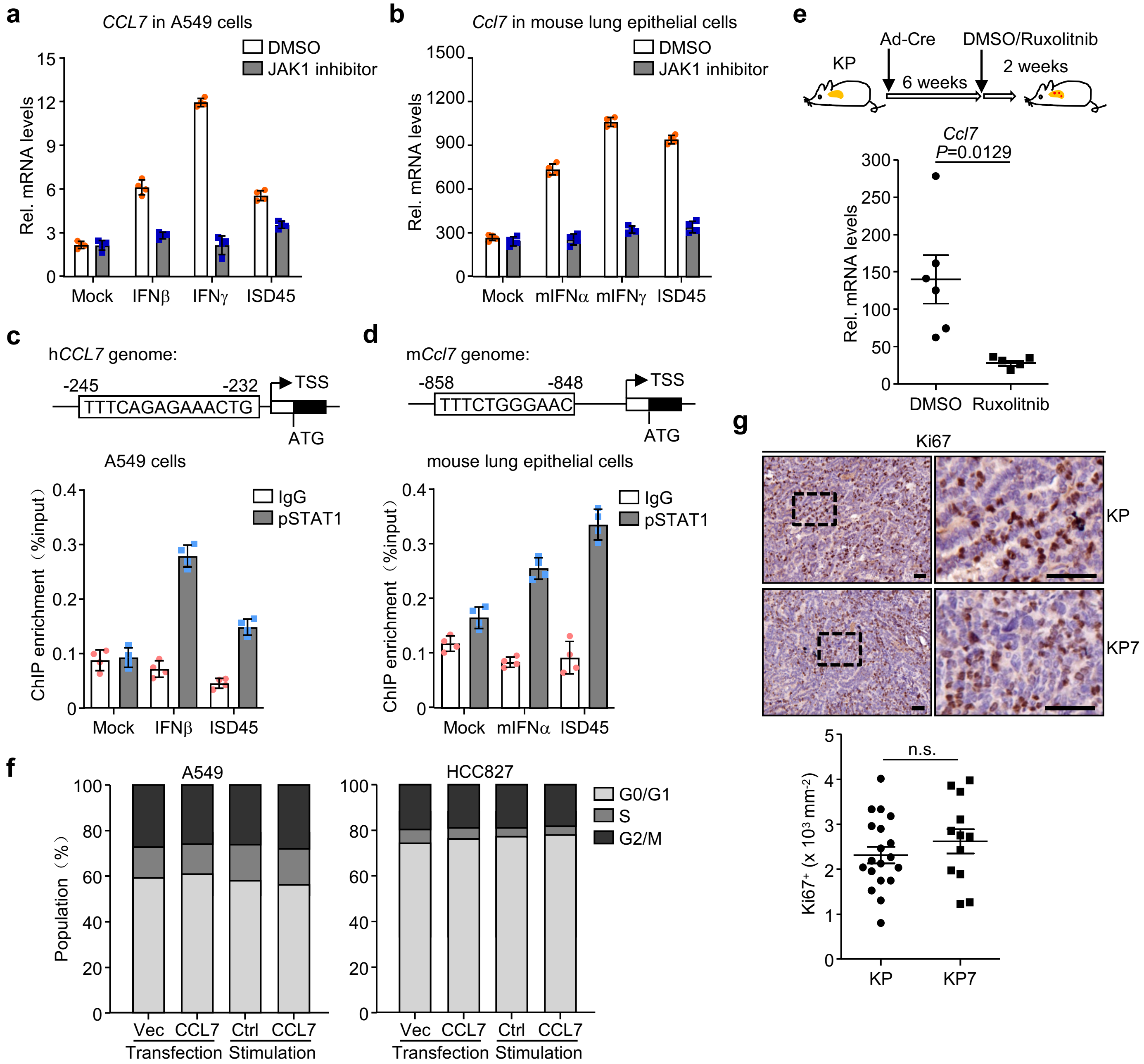

Supplementary Figure 3 Upregulation of CCL7 in NSCLC tumors is mediated by JAK-STAT pathways.

(**a**) qRT-PCR analysis of *CCL7* mRNA in A549 cells stimulated with IFNβ, IFNγ or transfected with ISD45 (n=4 technical replicates) for 6 h in the presence or absence of JAK1 inhibitor.

(**b**) qRT-PCR analysis of *Ccl7* mRNA in mouse lung epithelial stimulated with mIFNα, mIFNγ or transfected with ISD45 (n=4 technical replicates) for 6 h in the presence or absence of JAK1 inhibitor.

(**c**) Transcription factor binding site analysis of the sequence of *CCL7* promoter (upper scheme) and chromatin immunoprecipitation (ChIP) assay (lower graph) of the binding of pSTAT1 on *CCL7* promoter in A549 cells stimulated with IFNβ for 1 h or transfected with ISD45 (n=4 technical replicates) for 6 h.

(**d**) Transcription factor binding site analysis of the sequence of mouse *Ccl7* promoter (upper scheme) and ChIP assay of the binding of pSTAT1 on *Ccl7* promoter in mouse lung epithelial cells (lower graph) stimulated with mIFNα for 1 h or transfected with ISD45 (n=4 technical replicates) for 6 h.

(**e**) qRT-PCR analysis of *Ccl7* mRNA in tumor-burdened lungs of KP mice intranasally injected with Ad-Cre for 6 weeks followed by treatment of JAK1 inhibitor (n=5 independent mice) or DMSO (n=6 independent mice) for 2 weeks.

(**f**) Populations of G0/G1, S or G2/M stage of A549 or HCC827 cells transfected with empty vector or CCL7 or stimulated with or without CCL7 followed by PI staining and cell cycle analysis.

(**g**) IHC staining (upper images) and intensity analysis (lower graph) of Ki67 in tumor sections of KP (n=19) or KP7 (n=12) mice intranasally injected with Ad-Cre for 8 weeks.

Two-tailed student's *t*-test (**e, g**). n.s.: not significant. Scale bars, 50 μm. Graphs show mean ± SEM (**a-e, g**). Data are representative of two independent experiments (**a-f**) or combined results of four independent experiments (**g**). Source data are provided as a source data file.

Supplementary Figure 4

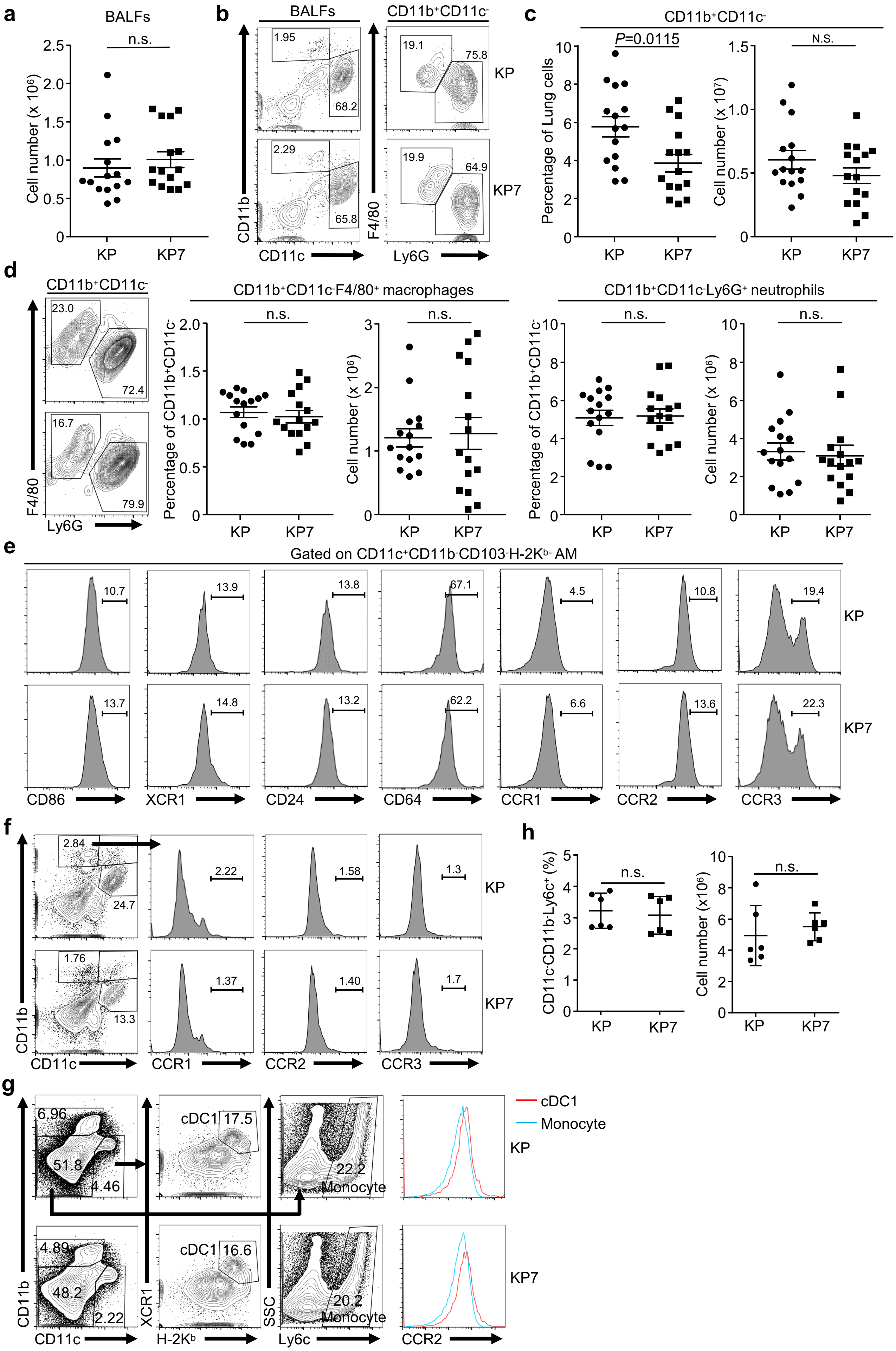

Supplementary Figure 4 Analysis of BALF and lung-infiltrated macrophages and neutrophils of KP or KP7 mice after tumor induction.

(**a**) Cell numbers of bronchoalveolar lavage fluids (BALFs) of KP (n=15) or KP7 (n=15) mice intranasally injected with Ad-Cre for 10 weeks.

(**b**) Flow cytometry analysis of BALFs from KP (n=15) or KP7 (n=15) mice treated as in (**a**).

(**c**) Percentages and numbers of $CD11b^{+}CD11c^{-}$ cells in tumor-burdened lungs of KP (n=15) or KP7 (n=15) mice treated as in (**a**).

(**d**) Flow cytometry analysis (left flow chart) and percentages and numbers of $CD11b^{+}CD11c^{-}F4/80^{+}$ macrophages (middle graph) and $CD11b^{+}CD11c^{-}Ly6G^{+}$ neutrophils (right graph) in tumor-burdened lungs of KP (n=15) or KP7 (n=15) mice treated as in (**a**).

(**e**) Flow cytometry analysis of the expression of CD86, XCR1, CD24, CD64, CCR1/2/3 on $CD11c^{+}CD11b^{-}CD103^{-}H\text{-}2K^{b-}$ AMs in tumor-burdened lungs of KP (n=15) or KP7 (n=15) mice treated as in (**a**).

(**f**) Flow cytometry analysis of the expression of CCR1/2/3 on $CD11c^{-}CD11b^{+}$ cells in tumor-burdened lungs of KP (n=15) or KP7 (n=15) mice treated as in (**a**).

(**g**) Flow cytometry analysis of the expression of $Ly6c^{+}$ monocytes in tumor-burdened lungs of KP (n=6) or KP7 (n=6) mice treated as in (**a**).

(**h**) Percentages and numbers of $CD11c^{-}CD11b^{-}Ly6c^{+}$ monocytes in in tumor-burdened lungs of KP (n=6) or KP7 (n=6) mice treated as in (**a**).

Two-tailed student's *t*-test (**a, c, d, h**). n.s.: not significant. Graphs show mean ± SEM (**a, c, d, h**). Data are combined results of three (**a-b**) or four (**c-f**) independent experiments or representative of two independent experiments (**g, h**). Source data are provided as a source data file.

Supplementary Figure 5

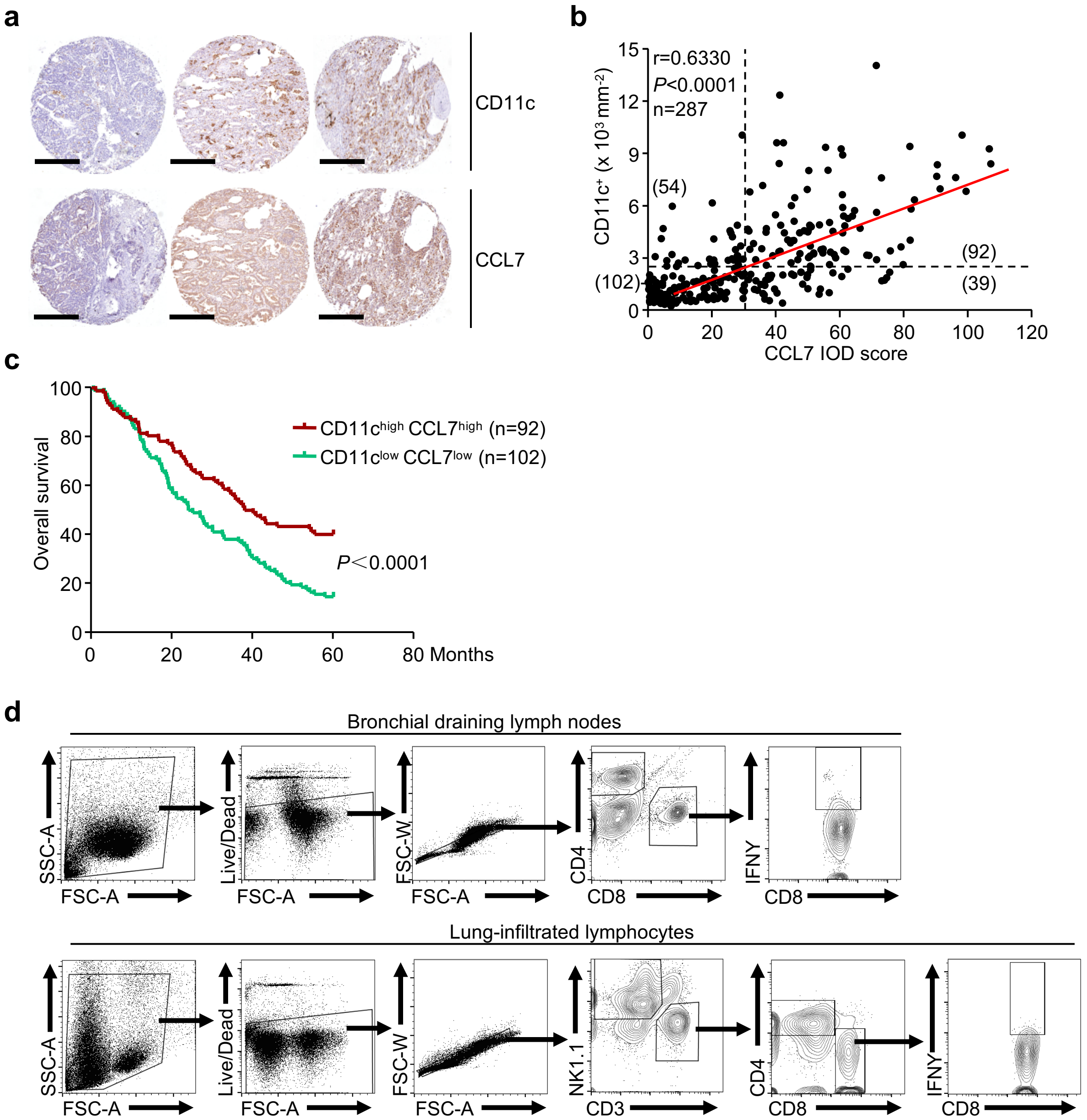

Supplementary Figure 5 CCL7 is positively correlated with CD11c in NSCLC tissues.

(**a**) Representative IHC staining of CD11c and CCL7 in tissue array of tumor biopsies from NSCLC patients (n=287).

(**b**) Pearson correlation analysis of CD11c and CCL7 IOD score of samples analyzed in (a).

(**c**) OS of NSCLC patients (n=92 and 102 for CD11c^{high} CCL7^{high} or CD11c^{low} CCL7^{low}, respectively) according to CCL7 and CD11c staining intensities.

(**d**) Gating strategy of bronchial draining lymph nodes and lung-infiltrated lymphocytes from tumor-baring KP (n=3) mice.

Log-Rank analysis (**c**). Scale bars, 500 μm.

Supplementary Figure 6

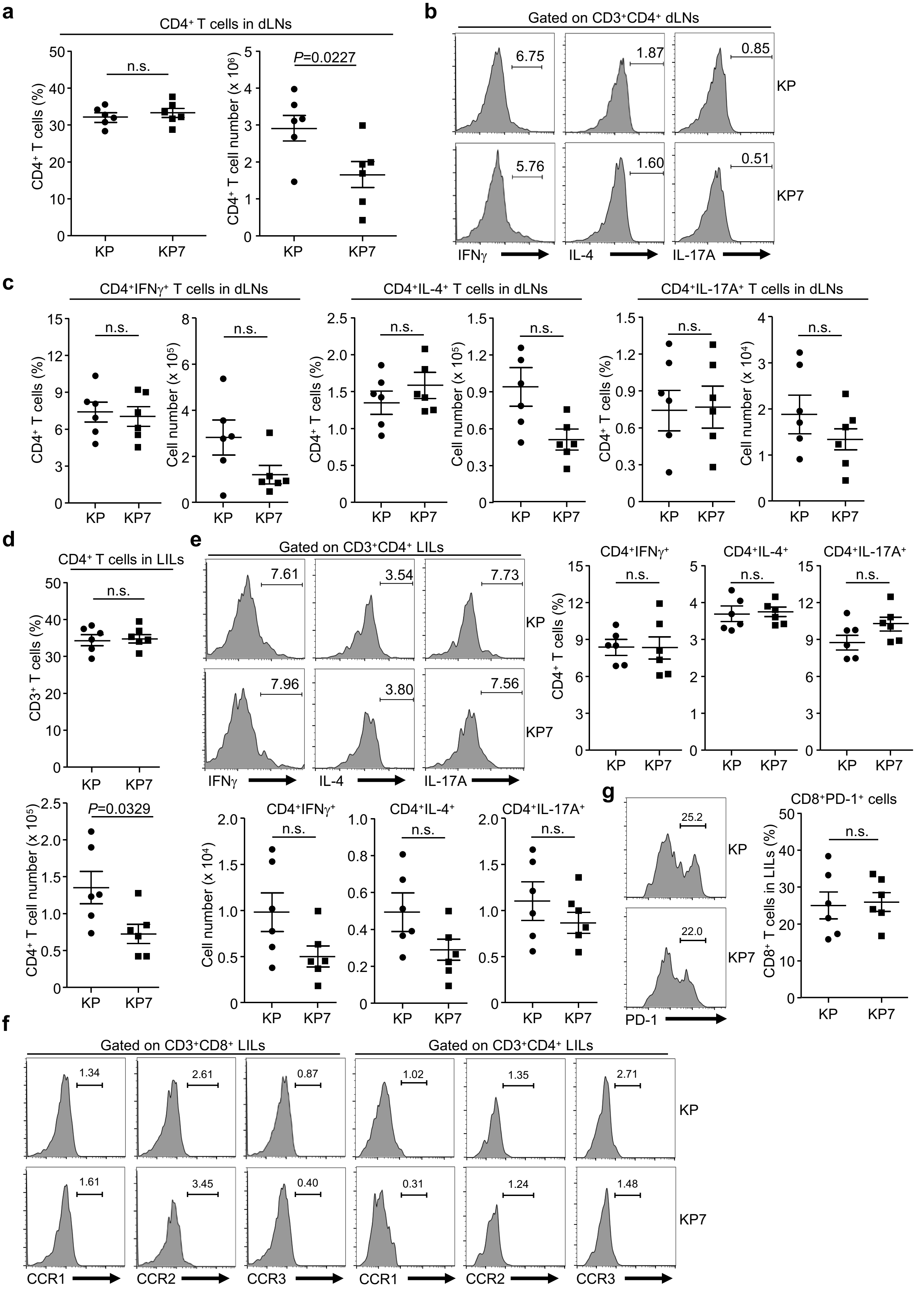

Supplementary Figure 6 CCL7 deficiency impairs $CD4^+$ T cell infiltration and expansion.
(**a**) Percentages and numbers of $CD3^+CD4^+$ T cells in bronchial draining LNs of KP (n=6) or KP7 (n=6) mice intranasally injected with Ad-Cre for 10 weeks.
(**b, c**) Flow cytometry analysis (**b**) and percentages and numbers (**c**) of $CD3^+CD4^+$ T cells that produce IFNγ, IL-4 or IL-17A in the bronchial draining LNs from mice treated as in (**a**).
(**d**) Percentages and numbers of $CD3^+CD4^+$ T cells in lung infiltrated lymphocytes (LIL) from tumor-burdened lungs of KP (n=6) or KP7 (n=6) mice treated as in (**a**).
(**e**) Flow cytometry analysis and percentages and numbers of $CD3^+CD4^+$ T cells that produce IFNγ, IL-4 or IL-17A in the LIL from mice treated as in (**a**).
(**f**) Flow cytometry analysis of CCR1, CCR2 and CCR3 on $CD3^+CD4^+$ or $CD3^+CD8^+$ T cells in LIL from tumor-burdened lungs of KP (n=6) or KP7 (n=6) mice treated as in (**a**).
(**g**) Flow cytometry analysis of PD-1 on $CD3^+CD8^+$ T cells in LIL from tumor-burdened lungs of KP (n=6) or KP7 (n=6) mice treated as in (**a**).
Two-tailed student's *t*-test (**a, c, d, e, g**). n.s.: not significant. Graphs show mean ± SEM (**a, c, d, e, g**). Data are combined results of two independent experiments. Source data are provided as a source data file.

Supplementary Figure 7

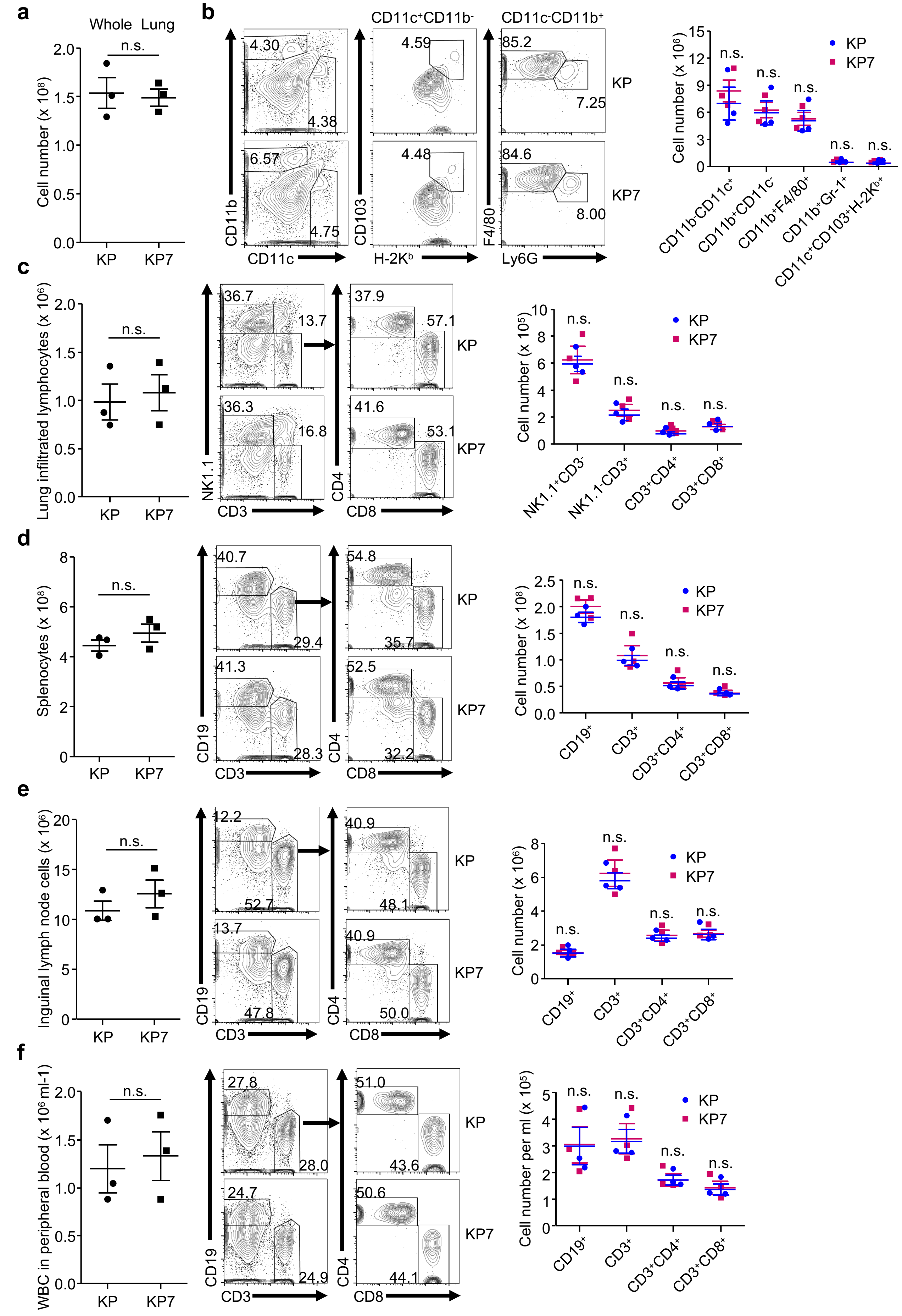

Supplementary Figure 7 CCL7 deficiency has minimal effect on immune cell homeostasis without challenge. Flow cytometry analysis of immune cells and quantitative data in lungs (**a, b**), lung infiltrated lymphocytes (**c**), spleen (**d**), peripheral lymph nodes (**e**) and peripheral blood (**f**) of 8-weeks old KP (n=3) and KP7 (n=3) mice. Two-tailed student's *t*-test. n.s.: not significant. Graphs show mean $\pm$ SEM. Data are representatives of two independent experiments. Source data are provided as a source data file.

Supplementary Figure 8

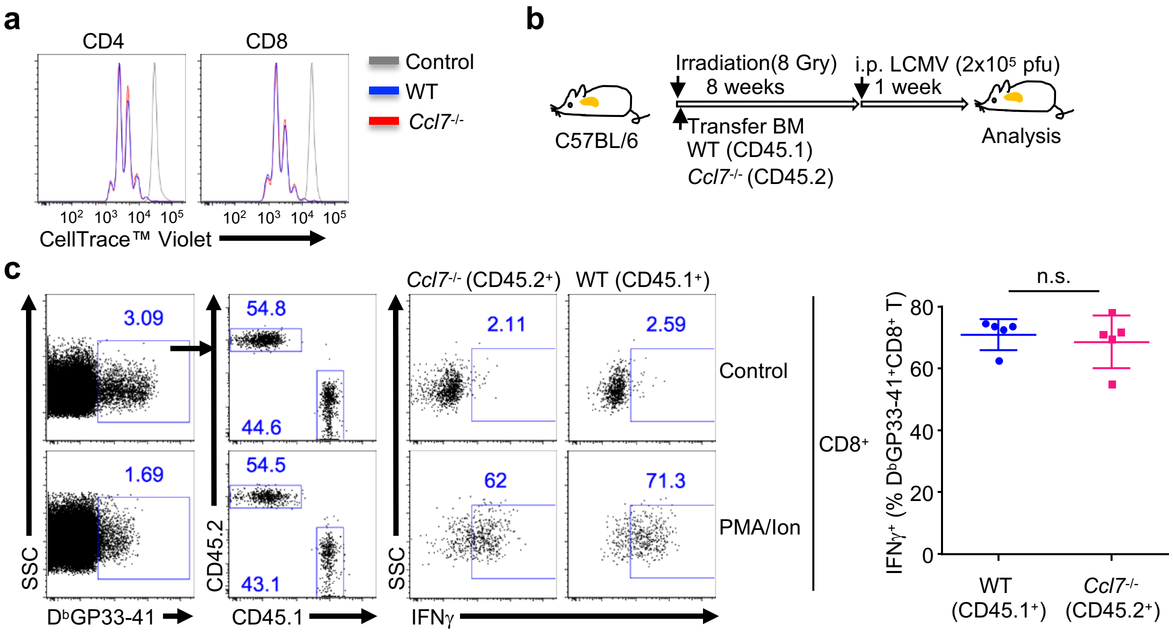

Supplementary Figure 8 CCL7 deficiency does not affect the differentiation or activation of T cells.
(**a**) Naive CD4+ or CD8+ T cells (CD62L^{+}CD44^{-}CD25^{-}) were sorted from wild-type (WT) or *Ccl7*$^{-/-}$ spleens, stained with CellTrace Violet and stimulated without (control) or with plate-bound anti-CD3/CD28 (0.5 μg/ml) for 72 hours followed by flow cytometry analysis.
(**b**) A scheme of bone marrow chimeric mice and LCMV infection.
(**c**) Flow cytometry (left flow charts) and statistical (right graph) analysis of splenocytes from mice (n=5) treated in (**b**).
Two-tailed student's *t*-test (**c**). n.s.: not significant. Graph show mean ± SD (**c**). Data are representatives of two independent experiments. Source data are provided as a source data file.

Supplementary Figure 9

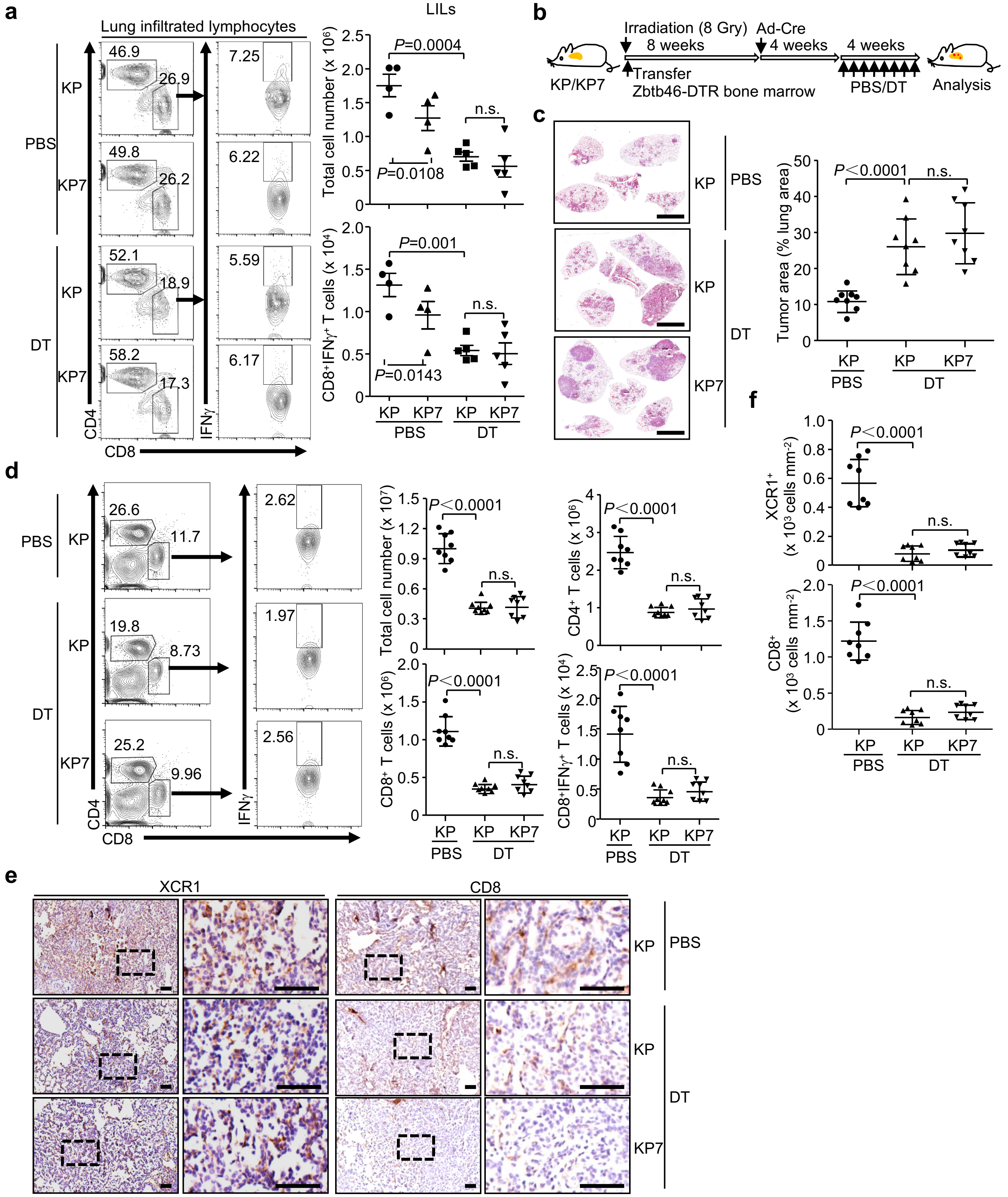

Supplementary Figure 9 Depletion of Zbtb46^{+} DCs promotes tumorigenesis in HP or KP7 mice.
(**a**) Flow cytometry analysis and quantitative data of lung infiltrated lymphocytes of irradiated KP (n=4 and 5 for PBS and DT, respectively) or KP7 (n=4 and 5 for PBS and DT, respectively) mice that were transferred with CD11c-DTR bone marrow cells for 8 weeks followed by intranassally injection of Ad-Cre and intraperitoneal injection of PBS or DT.
(**b**) A scheme of Zbtb46-DTR bone marrow transfer and tumor induction in KP or KP7 mice. The KP or KP7 mice were irradiated followed by transfer of Zbtb46-DTR bone marrow cells ($1x10^{6}$ per mouse). Eight weeks later, the mice were intranassally injected with Ad-Cre. Five weeks later, the mice were intraperitoneally injected with PBS or DT every other day for 4 weeks followed by various analysis.
(**c**) HE staining and tumor areas in KP (n=8 for PBS or DT) or KP7 (n=8) mice treated as in (**b**).
(**d**) Flow cytometry analysis of cells in bronchial draining LN from KP (n=8 for PBS or DT) or KP7 (n=8) mice treated as in (**b**).
(**e, f**) IHC staining (e) and quantification analysis (f) of XCR1 and CD8 in KP (n=8 for PBS or DT) or KP7 (n=8) mice treated as in (**b**).
Two-tailed student's *t*-test (**a, c, d, f**). n.s.: not significant. Scale bars represent 5 mm (**c**) and 50 μm (**e**), respectively. Graphs show mean ± SEM (**a, c, d, f**). Data are representative (**a**) or combination (**c-f**) of two independent experiments. Source data are provided as a source data file.

Supplementary Figure 10

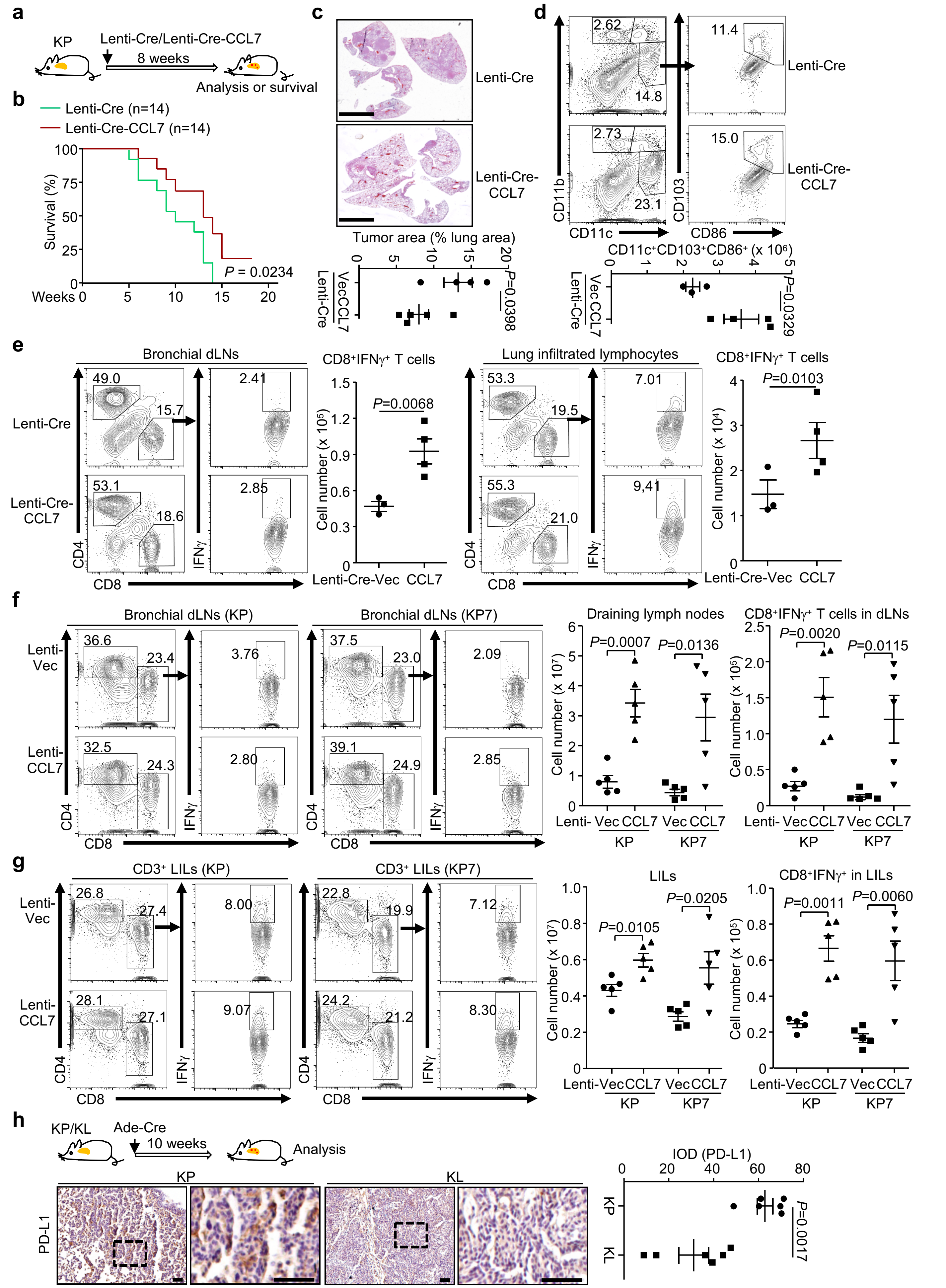

Supplementary Figure 10 Administration of CCL7 inhibits tumorigenesis in KP mice.
(**a**) A scheme of administration of CCL7 into KP mice. KP mice were intranasally infected with Lenti-Cre or Lenti-Cre-CCL7 (2.0 x 10^6 pfu/mouse) for 8 weeks or for survival observation.
(**b**) Kaplan-Meier survival analysis of KP mice intranassaly injected with Lenti-Cre (n=14) or Lenti-Cre-CCL7 (n=14) (2.0 x 10^6 pfu/mouse).
(**c**) HE-stained lung sections and tumor burden analysis of KP (n=4 or 5 for Vec or CCL7, respectively) mice treated as in (**a**).
(**d**) Flow cytometry analysis of $CD11c^+$ DCs in tumor-burdened lungs from KP mice intranasally injected with Lenti-Cre (n=3) or Lenti-Cre-CCL7 (n=4) for 10 weeks.
(**e**) Flow cytometry analysis of lymphocytes in bronchial dLNs (left flow charts and graph) or lung infiltrated lymphocytes (LILs) (right flow charts and graph) of KP (n=3 or 4 for Vec or CCL7, respectively) mice treated as in (**d**).
(**f**) Flow cytometry analysis (lest flow charts) and cell numbers (right graphs) of bronchial dLNs of KP (n=5 for Vec or CCL7) or KP7 (n=5 for Vec or CCL7) mice intranasally injected with Ad-Cre for 6 weeks followed by intranasal injection of Lenti-Vec or Lenti-CCL7 for 5 weeks.
(**g**) Flow cytometry analysis (left flow charts) and cell numbers (right graphs) of $CD3^+$ LILs of KP (n=5 for Vec or CCL7) or KP7 (n=5 for Vec or CCL7) mice treated as in (**f**).
(**h**) A scheme of tumor induction of KP or KL mice that were intranasally injected with Ad-Cre (1 x 10^6) for 10 weeks (upper). IHC and IOD analysis of PD-L1 expression in lung tumors from KP (n=6) or KL (n=6) mice at 10 weeks after tumor induction (lower images and graph).
Log-rank test (**b**) or two-tailed student's *t*-test (**c-h**). Scale bars, 5 mm (c) or 50 μm (h), respectively. Graph show mean $\pm$ SEM (**c-h**). Data are combined results of two (**b**) or representatives of two (**c-h**) independent experiments. Source data are provided as a source data file.

Supplementary Table 1 Clinical information of NSCLC patient in Cohort 1

No.	Genger	Age(years)	pTpNpM Status	*Ccl7* (Tumor)	*Ccl7* (Normal)
1	M	58	T4N0M0	2.535	0.000
2	F	79	T2aN0M0	3.801	1.826
3	M	63	T2N2M0	11.726	4.504
4	F	67	T2aN0M0	0.019	0.073
5	M	61	T2bN1M0	0.417	0.000
6	M	56	T2N2M0	6.127	0.552
7	M	68	T4N0M0	12.043	0.000
8	M	49	T2bN2M0	2.114	1.184
9	M	63	T2N2M0	1.473	1.095
10	F	58	M1	1.055	3.184
11	M	74	T2bN0M0	12.571	0.255
12	M	62	T2bN0M0	2.093	1.196
13	M	72	T2aN0M0	9.421	0.000
15	M	49	T4N0M1	1.233	0.000
14	M	66	T2bN0M0	1.797	0.243
16	M	62	T2aN0M0	0.804	0.000
17	F	48	M1	5.226	1.340
18	M	71	T2N0M0	0.188	0.956

Supplementary Table 2 Clinical information of NSCLC patient in Cohort 2

No.	Genger	Age(years)	pTpNpM Status	*Ccl7* (Tumor)	*Ccl7* (Normal)
1	M	64	T2aN2M0	12.62	0.00
2	M	59	T2bN1M0	0.07	0.00
3	M	54	T2N2M0	9.65	0.00
4	F	68	T2N2M0	0.11	0.00
5	M	57	T2N0M0	0.23	0.00
6	F	52	T2N2M0	0.11	0.00
7	M	66	T2N0M0	6.06	0.09
8	M	61	T2N1M0	3.36	0.00
9	F	51	T2N2M1	1.17	0.02
10	M	53	T4N1M0	5.87	0.12
11	M	60	T2aN1M0	0.16	0.00
12	M	65	T1N0M0	0.76	0.55
13	F	49	T2N2M0	12.40	8.54
15	M	65	T2N2M0	5.21	0.00
14	F	57	T3N0M0	1.65	0.00
16	M	56	T2aN0M0	1.38	0.00
17	M	51	T2N2M0	0.21	0.00
18	F	54	T2N0M0	0.44	0.00
19	F	34	T2N3M0	32.95	0.00
20	M	66	T2aN0M0	5.28	0.38
21	F	49	T2aN0M0	24.30	11.27
22	F	61	T1bN0M0	0.00	0.00
23	M	59	T2aN0M0	0.90	0.00
24	M	72	T2aN0M0	2.69	0.55
25	M	60	T3N3M0	2.99	0.00
26	M	48	T2aN0M0	0.36	0.00
27	F	70	T2aN0M0	0.00	0.00
28	M	56	T1N1M0	0.06	0.00
29	M	50	T2aN0M0	0.00	0.03
30	M	56	T1N1M0	0.28	0.89
31	F	77	T2aN0M0	9.50	4.57
32	M	61	T2N2M0	29.32	11.26
33	M	66	T4N0M0	3.01	0.00
34	M	47	T2bN2M0	0.53	0.00
35	F	56	T4N2M1	0.26	0.00
36	M	72	T2bN0M0	3.14	0.64
37	F	43	T2aN2M0	0.00	7.03
38	M	47	T4N0M1	3.08	0.00
39	M	64	T2bN0M0	4.49	0.61
40	M	60	T2aN0M0	2.01	0.00
41	M	69	T2N0M0	0.00	0.01
42	F	29	M1	13.07	3.35
43	M	71	T2N0M0	20.61	0.11
44	M	67	T2N0M0	0.00	2.39

Supplementary Table 3 Summary of NSCLC patients in Cohort 2

Vaviables	All cases(n=44)	CCL7 relative mRNA level		P value
		Low（＜2）	High（≥2）	
Age（years）				0.6926
≤56	19	12	7	
＞56	25	11	14	
Gender				0.3864
Male	30	14	16	
Female	14	9	5	
Pathologic type				0.4138
Squamous carcinoma	18	8	10	
Adenocarcinoma	26	15	11	
Degree differentiation				0.7647
well	10	6	4	
poor	34	17	17	
Smoking				0.6936
Yes	27	11	16	
No	17	12	5	
pT Status				0.1156
pT1	4	4	0	
pT2	33	17	16	
pT3	2	1	1	
pT4	5	1	4	
pN Status				0.3979
pN-	23	12	11	
pN+	21	11	10	
pM Status				0.8442
pM-	40	21	19	
pM+	4	2	2	
UICC				**0.0472**
I	16	9	7	
II	10	7	3	
III	14	5	9	
IV	4	2	2	

Supplementary Table 4 Summary of NSCLC patients in Cohort 3

Vaviables	All cases(n=287)	CCL7 expression		P value
		Low（<30）	High（IOD≥30）	
Age（years）				0.4938
≤56	80	38	42	
＞56	207	103	101	
Gender				0.8351
Male	178	88	90	
Female	109	53	56	
Pathologic type				**0.0018**
Squamous carcinoma	29	7	22	
Adenocarcinoma	258	134	124	
Degree differentiation				0.144
well	103	46	57	
poor	184	95	89	
5 years survival				**0.0073**
Life	79	25	54	
Death	208	116	92	
pT Status				0.7425
pT1	51	23	28	
pT2	141	72	69	
pT3	50	26	24	
pT4	45	24	21	
pN Status				0.6754
pN-	103	51	52	
pN+	184	94	90	
pM Status				0.8276
pM-	235	118	117	
pM+	52	27	25	
UICC				0.4154
I	62	29	33	
II	56	32	24	
III	122	59	83	
IV	48	25	23	

Supplementary Table 5 Clinical information of NSCLC patient in Cohort 4

No.	Pathologic type	pTpNpM Status	UICC	Therapeutic schedule	Effect	PD-L1 TPS(%)	CD11C	CCL7 IOD
1	SC	M1	Ⅳ	Keytruda + Albumin paclitaxel	PR	30%	38.27	88.27
2	AC	M1	Ⅳ	Keytruda + Pemetrexed + Platinum	PR	30%	34.17	190.52
3	AC	M1	Ⅳ	Keytruda + Pemetrexed + Platinum	PR	50%	30.57	29.98
4	SC	T4N0M0	ⅢA	Keytruda + Albumin paclitaxel + Platinum	PR	60%	16.59	211.22
5	SC	M1	Ⅳ	Sintilimab + Docetaxel	PR	<1%	31.63	119.13
6	AC	M1	Ⅳ	Keytruda + Pemetrexed + Platinum	PR	–	29.18	42.39
7	AC	M1	Ⅳ	Keytruda + Pemetrexed + Platinum	PR	10%	25.09	62.46
8	SC	T4N2M0	ⅢB	Keytruda + Albumin paclitaxel + Platinum	PR	3%	30.16	135.99
9	AC	T3N2M0	ⅢA	Sintilimab + Albumin paclitaxel	PR	<1%	40.31	90.39
10	AC	T4N3M1	Ⅳ	Keytruda + Albumin paclitaxel	PR	5%	2.15	101.17
11	AC	T4N2M1	Ⅳ	Keytruda + Pemetrexed + Platinum	PR	<1%	34.49	134.36
12	SC	M1	Ⅳ	Sintilimab + Albumin paclitaxel	PR	10%	79.07	50.56
13	AC	M1	Ⅳ	Sintilimab + Pemetrexed + Platinum	PR	<1%	26.17	142.62
15	AC	T4N3M1	Ⅳ	Keytruda + Pemetrexed + Platinum	PR	5%	12.57	133.62
14	AC	M1	Ⅳ	Sintilimab + Pemetrexed + Platinum	PR	3%	32.86	110.54
16	AC	M1	Ⅳ	Keytruda + Pemetrexed + Platinum	PR	5%	23.62	18.50
17	SC	T3N2M1a	Ⅳ	Sintilimab + Albumin paclitaxel	SD	60%	44.72	210.74
18	AC	T2N3M1	Ⅳ	Keytruda + Pemetrexed + Platinum	SD	–	31.86	149.48
19	SC	T2N3M0	ⅢB	Keytruda + Albumin paclitaxel	SD	–	7.73	26.79
20	SC			Keytruda + Albumin paclitaxel	SD	3%	41.75	122.43
21	AC	M1	Ⅳ	Keytruda + Albumin paclitaxel	SD	7%	36.83	78.95
22	AC	T3N3M0	ⅢB	Keytruda + Navelbine	SD	60%	20.78	134.02
23	AC	T3N2M0	ⅢB	Keytruda + Albumin paclitaxel	SD	70-80%	40.02	58.21
24	AC	T2bN1M0	ⅡB	Keytruda + Pemetrexed	SD	70%	56.17	156.98
25	SC	T3N0M1	Ⅳ	Sintilimab + Docetaxel	SD	3%	12.64	108.78
26	AC	T3N2M0	ⅢB	Sintilimab + Docetaxel + Platinum	SD	5%	18.22	50.56
27	AC			Keytruda + Albumin paclitaxel + Platinum	SD	<1%	57.74	122.30
28	AC	T2N2M1	Ⅳ	Sintilimab + Albumin paclitaxel	SD	10%	53.36	56.83
29	AC	T2N2M1	Ⅳ	Sintilimab + Pemetrexed + Cisplatin	SD	5%	40.31	5.57
30	AC	T4N1M1	Ⅳ	Sintilimab + Albumin paclitaxel	PD	<1%	6.53	47.15
31	AC	T3N2M1	Ⅳ	Keytruda + Pemetrexed + Platinum	PD	50%	10.22	28.07
32	AC	M1	Ⅳ	Sintilimab + Albumin paclitaxel	PD	5%	0.29	17.31
33	AC	M1	Ⅳ	truda + Tegafur, gimeracil and oteracil potas	PD	30%	2.62	54.52
34	AC	T1bN3M1	Ⅳ	Keytruda + Albumin paclitaxel + Nedaplatin	PD	70%	4.04	8.41
35	AC	T1N3M1	Ⅳ	Keytruda + Docetaxel + Carboplatin	PD	90%	15.74	45.43

Supplementary Table 6 Primer sequences.

Name	Forward (5' to 3')	Reverse (5' to 3')
GAPDH	ACGGCCAGGTCATCACTATT	TGGCATAGAGGTCTTTACGGA
CCL7	GTCCCCGGGAAGCTGTAATC	GCTTTGGAGTTTGGGTTTTCTT
m*β-Actin*	ACGGCCAGGTCATCACTATT	TGGCATAGAGGTCTTTACGGA
m*Ccl7*	TCAAGAGCTACAGAAGGATCACC	TGGAGTTGGGGTTTTCATGTCT
m*Ifng*	CAGCAACAGCAAGGCGAAAAAGG	TTTCCGCTTCCTGAGGCTGGAT
h*CCL7* promoter	GGTATCCCTGATTCCTTCCTTT	GTGGTAGGTGAGGTAGGGTCTA
m*Ccl7* promoter	CAATATTTCTGGGAACAGAA	CACATGATGCCTTAAAGTGT
Ccl7IRES-ZsGreen (WT/KI)	ATCCATTCCTGGGTCATGTCATC	
Ccl7IRES-ZsGreen (KI)		ACTTCATGGTCATCTCCTTGGTCA
Ccl7IRES-ZsGreen (WT)		CAAATCACACCAAAGTACATGGC

Supplementary Table 7 A list of antibodies.

REAGENT or RESOURCE	SOURCE	IDENTIFIER	DILUTION
Anti-human/mouse CCL7	Sungenebiotech	Cat# E0728	1:500
Anti-human CD11c (D3V1E)	CST	Cat# 45581	1:500
Anti-mouse CD11c (D1V9Y)	CST	Cat# 97585	1:500
Anti-mouse CD103 (2E7)	BioLegend	Cat# 121401	1:500
Anti-mouse CD8a (D4W2Z)	CST	Cat# 98941	1:500
Anti-mouse PD-L1 (E1L3N)	CST	Cat# 13684	1:500
Anti-mouse Ki67	Abclonal	Cat# A11907	1:500
Anti-human pSTAT1 (58D6)	CST	Cat# 9167S	1:500
Rabbit lgG	Santa	Cat# SC2025	1:500
CD16/32 (93)	BioLegend	Cat# 101302	1:200
Anti-mouse CD11c (N418) PerCP	BioLegend	Cat# 117324	1:200
Anti-mouse CD11c (N418) APC	BioLegend	Cat# 117310	1:200
Anti-mouse CD11b (M1/70) FITC	BioLegend	Cat# 101206	1:200
Anti-mouse CD11b (M1/70) PE	BioLegend	Cat# 101208	1:200
Anti-mouse CD103 (2E7) BV510	BioLegend	Cat# 121423	1:200
Anti-mouse H-2K^b (M5/114) BV421	BioLegend	Cat# 107632	1:200
Anti-mouse CD86 (GL-1) FITC	BioLegend	Cat# 105005	1:200
Anti-mouse CD8 (53-6.7) PE	BioLegend	Cat# 100708	1:200
Anti-mouse CCR1 (S15040E) FITC	BioLegend	Cat# 152505	1:200
Anti-mouse CCR2 (SA203G11) BV510	BioLegend	Cat# 150617	1:200
Anti-mouse CCR3 (J073E5) BV421	BioLegend	Cat# 144517	1:200
Anti-mouse F4/80 (BM8) PE	BioLegend	Cat# 123110	1:200
Anti-mouse Ly6G (1A8) APC-Cy7	BioLegend	Cat# 127623	1:200
Anti-mouse NK1.1 (PK136) BV421	BioLegend	Cat# 108731	1:200
Anti-mouse CD3 (145-2C11) APC-Cy7	BioLegend	Cat# 100330	1:200
Anti-mouse CD4 (GK1.5) APC	BioLegend	Cat# 100411	1:200
Anti-mouse CD8 (53-6.7) FITC	BioLegend	Cat# 100706	1:200
Anti-mouse IFNg (XMG1.2) PE	BioLegend	Cat# 505807	1:200
Anti-mouse IL-4 (11B11) PE	BioLegend	Cat# 504104	1:200
Anti-mouse IL-17A (TC11-18) FITC	BioLegend	Cat# 506908	1:200
Anti-mouse PD-1 (RMP1-30) PE	BioLegend	Cat# 109104	1:200
Anti-mouse PD-1 (J43)	Bio X cell	Cat# BE0033-2	used for IP injection
Hamster lgG	Bio X cell	Cat# BE0091	used for IP injection
