# Supplementary material for: CCL7 recruits cDC1 to promote antitumor immunity and facilitate checkpoint immunotherapy to non-small cell lung cancer
Source: Nat Commun. 2020 Nov 30;11:6119. doi: 10.1038/s41467-020-19973-6 (PMC7704643; doi:10.1038/s41467-020-19973-6)
Supplement: Supplementary file 2 — Descriptions of Additional Supplementary Files [file 41467_2020_19973_MOESM2_ESM.pdf]

**Descriptions of Additional Supplementary Files**

**Supplementary Data 1**

**Description:** Clinical information of NSCLC patient in Cohort 3
