# Supplementary material for: CCL7 recruits cDC1 to promote antitumor immunity and facilitate checkpoint immunotherapy to non-small cell lung cancer
Source: Nat Commun. 2020 Nov 30;11:6119. doi: 10.1038/s41467-020-19973-6 (PMC7704643; doi:10.1038/s41467-020-19973-6)
Supplement: Supplementary file 4 — Reporting Summary [file 41467_2020_19973_MOESM4_ESM.pdf]

nature research

Corresponding author(s): Bo Zhong

Last updated by author(s): Oct 27, 2020

Reporting Summary

Nature Research wishes to improve the reproducibility of the work that we publish. This form provides structure for consistency and transparency in reporting. For further information on Nature Research policies, see our Editorial Policies and the Editorial Policy Checklist.

Statistics

For all statistical analyses, confirm that the following items are present in the figure legend, table legend, main text, or Methods section.

n/a	Confirmed	
☐	☒	The exact sample size (*n*) for each experimental group/condition, given as a discrete number and unit of measurement
☐	☒	A statement on whether measurements were taken from distinct samples or whether the same sample was measured repeatedly
☐	☒	The statistical test(s) used AND whether they are one- or two-sided<br>*Only common tests should be described solely by name; describe more complex techniques in the Methods section.*
☒	☐	A description of all covariates tested
☒	☐	A description of any assumptions or corrections, such as tests of normality and adjustment for multiple comparisons
☐	☒	A full description of the statistical parameters including central tendency (e.g. means) or other basic estimates (e.g. regression coefficient) AND variation (e.g. standard deviation) or associated estimates of uncertainty (e.g. confidence intervals)
☐	☒	For null hypothesis testing, the test statistic (e.g. *F*, *t*, *r*) with confidence intervals, effect sizes, degrees of freedom and *P* value noted<br>*Give P values as exact values whenever suitable.*
☒	☐	For Bayesian analysis, information on the choice of priors and Markov chain Monte Carlo settings
☐	☒	For hierarchical and complex designs, identification of the appropriate level for tests and full reporting of outcomes
☐	☒	Estimates of effect sizes (e.g. Cohen's *d*, Pearson's *r*), indicating how they were calculated

Our web collection on statistics for biologists contains articles on many of the points above.

Software and code

Policy information about availability of computer code

Data collection	BD FACSDiVa Software v8.0.1.1 for FACS<br>Bio-Rad CFX Manager 3.1 for qRT-PCR.<br>Aperio imagescope (v12.3.2.8013) installed on a Leica Aperio VERSA 8 Brightfield, Fluorescence & FISH Digital Pathology Scanner for immunohistochemistry and HE sides imaging.
Data analysis	Prism 6 for graphs and statistical analysis.<br>Flowjo 10.6.2 for FACS plots.<br>Image-Pro Plus 6.0 for immunohistochemistry signal quantification.

For manuscripts utilizing custom algorithms or software that are central to the research but not yet described in published literature, software must be made available to editors and reviewers. We strongly encourage code deposition in a community repository (e.g. GitHub). See the Nature Research guidelines for submitting code & software for further information.

Data

Policy information about availability of data

All manuscripts must include a data availability statement. This statement should provide the following information, where applicable:

- Accession codes, unique identifiers, or web links for publicly available datasets
- A list of figures that have associated raw data
- A description of any restrictions on data availability

Source data are provided with this paper. The source data underlying Figs. 1a-c, 2b-f, 3b-d, f, 4, 5b, d-g, 6c-g, 7b, c, e and 8 and Supplementary Figs. 1, 2a, 3, 4a, c, d, h, 6a, c-g, 7, 8c, 9 and 10 are provided as a Source Data file. All the other data supporting the findings of this study are available within the article and its supplementary information files and from the corresponding author upon reasonable request. A reporting summary for this article is available as a Supplementary

Information file.

Field-specific reporting

Please select the one below that is the best fit for your research. If you are not sure, read the appropriate sections before making your selection.

☒ Life sciences ☐ Behavioural & social sciences ☐ Ecological, evolutionary & environmental sciences

For a reference copy of the document with all sections, see nature.com/documents/nr-reporting-summary-flat.pdf

Life sciences study design

All studies must disclose on these points even when the disclosure is negative.

Sample size	The group sizes of the animals chosen are based on the numbers we used for previous publications (Liu X et al., Nature, 2019, 567: 525-529; Wang XM et al., Nat Cancer, 2020, 1: 811-825). For non-animal experiments, technical replicates (n=4) were performed and the experiments were repeated at least two independent times.
Data exclusions	No data was excluded from our study.
Replication	The experiments were performed with 2-4 independent replications. The replication numbers were described in the corresponding figure legends. For each replication, age- and sex-matched mice were used. For qRT-PCR with cells, four technical replications were performed and the experiments were independently repeated at least two times.
Randomization	Age- and sex-matched mice were selected and included in different groups. Selection of mice was based on genotype without a plan for randomization. Cells of different genotypes were seeded in plates and randomly treated with various stimuli for different time points followed by qRT-PCR, ChIP or proliferation analysis.
Blinding	Data collection of most mouse experiments were performed in a blinding manner. Age- and sex-matched mice of different genotypes were allocated to a same cage (3-6 mice in each cage) and treated by some of the authors. At the end of the study, other authors collected and analyzed the data and numbered the data according to the ear tag numbers. Because cells were treated with different stimuli with a label on the plate which was known to the experiment performers, cellular and biochemical experiments were not performed in a blinding manner.

Reporting for specific materials, systems and methods

We require information from authors about some types of materials, experimental systems and methods used in many studies. Here, indicate whether each material, system or method listed is relevant to your study. If you are not sure if a list item applies to your research, read the appropriate section before selecting a response.

Materials & experimental systems

n/a	Involved in the study	
☐	☒	Antibodies
☐	☒	Eukaryotic cell lines
☒	☐	Palaeontology and archaeology
☐	☒	Animals and other organisms
☐	☒	Human research participants
☒	☐	Clinical data
☒	☐	Dual use research of concern

Methods

n/a	Involved in the study	
☒	☐	ChIP-seq
☐	☒	Flow cytometry
☒	☐	MRI-based neuroimaging

Antibodies

Antibodies used

REAGENT or RESOURCE SOURCE IDENTIFIER
Antibodies
name provider cat# clone lot dilution
Anti-human/mouse CCL7 Sungenebiotech Cat# E0728 1:500
Anti-human CD11c (D3V1E) CST Cat# 45581 1:500
Anti-mouse CD11c (D1V9Y) CST Cat# 97585 1:500
Anti-mouse CD103 (2E7) BioLegend Cat# 121401 1:500
Anti-mouse CD8a (D4W2Z) CST Cat# 98941 1:500
Anti-mouse PD-L1 (E1L3N) CST Cat# 13684 1:500
Anti-mouse Ki67 Abclonal Cat# A11907 1:500
Anti-human pSTAT1 (58D6) CST Cat# 9167S 1:500
Rabbit lgG Santa Cat# SC2025 1:500
CD16/32 (93) BioLegend Cat# 101302 1:200
Anti-mouse CD11c (N418) PerCP BioLegend Cat# 117324 1:200

Anti-mouse CD11c (N418) APC BioLegend Cat# 117310 1:200
Anti-mouse CD11b (M1/70) FITC BioLegend Cat# 101206 1:200
Anti-mouse CD11b (M1/70) PE BioLegend Cat# 101208 1:200
Anti-mouse CD103 (2E7) BV510 BioLegend Cat# 121423 1:200
Anti-mouse H-2Kb (M5/114) BV421 BioLegend Cat# 107632 1:200
Anti-mouse CD86 (GL-1) FITC BioLegend Cat# 105005 1:200
Anti-mouse CD8 (53-6.7) PE BioLegend Cat# 100708 1:200
Anti-mouse CCR1 (S15040E) FITC BioLegend Cat# 152505 1:200
Anti-mouse CCR2 (SA203G11) BV510 BioLegend Cat# 150617 1:200
Anti-mouse CCR3 (J073E5) BV421 BioLegend Cat# 144517 1:200
Anti-mouse F4/80 (BM8) PE BioLegend Cat# 123110 1:200
Anti-mouse Ly6G (1A8) APC-Cy7 BioLegend Cat# 127623 1:200
Anti-mouse NK1.1 (PK136) BV421 BioLegend Cat# 108731 1:200
Anti-mouse CD3 (145-2C11) APC-Cy7 BioLegend Cat# 100330 1:200
Anti-mouse CD4 (GK1.5) APC BioLegend Cat# 100411 1:200
Anti-mouse CD8 (53-6.7) FITC BioLegend Cat# 100706 1:200
Anti-mouse IFNg (XMG1.2) PE BioLegend Cat# 505807 1:200
Anti-mouse IL-4 (11B11) PE BioLegend Cat# 504104 1:200
Anti-mouse IL-17A (TC11-18) FITC BioLegend Cat# 506908 1:200
Anti-mouse PD-1 (RMP1-30) PE BioLegend Cat# 109104 1:200
Anti-mouse PD-1 (J43) Bio X cell Cat# BE0033-2 (used for IP injection)
Hamster lgG Bio X cell Cat# BE0091 (used for IP injection)

Validation

The antibodies have been validated according to the online information.
name species provider cat# application website
Anti-CCL7 human/mouse Sungenebiotech Cat# E0728 WB, IHC, This study.
Anti-CD11c (D3V1E) human CST Cat# 45581 WB, IHC, IF https://www.cellsignal.cn/products/primary-antibodies/cd11c-d3v1e-xp-rabbit-mab/45581?N=4294956287&Ntt=45581&fromPage=plp
Anti-CD11c (D1V9Y) mouse CST Cat# 97585 WB, IHC, IF https://www.cellsignal.cn/products/primary-antibodies/cd11c-d1v9y-rabbit-mab/97585?N=4294956287&Ntt=97585&fromPage=plp
Anti-CD103 (2E7) BioLegend Cat# 121401 IP, IHC https://www.biolegend.com/en-us/products/purified-anti-mouse-cd103-antibody-3572
Anti-CD8a (D4W2Z) mouse CST Cat# 98941 WB, IHC https://www.cellsignal.cn/products/primary-antibodies/cd8a-d4w2z-xp-rabbit-mab-mouse-specific/98941?N=4294956287&Ntt=98941&fromPage=plp
Anti-PD-L1 (E1L3N) mouse CST Cat# 13684 WB, IHC, IP, Flow https://www.cellsignal.cn/products/primary-antibodies/pd-l1-e1l3n-xp-rabbit-mab/13684?N=4294956287&Ntt=13684&fromPage=plp
Anti-Ki67 Abclonal mouse Cat# A11907 WB, IHC, IF, ICC https://abclonal.com.cn/Datasheet/Antibodies/A11907.pdf
Anti-pSTAT1 (58D6) human CST Cat# 9167S WB, IHC, IP ,Flow, IF, Chip https://www.cellsignal.cn/products/primary-antibodies/phospho-stat1-tyr701-58d6-rabbit-mab/9167?N=4294956287&Ntt=9167s&fromPage=plp
Rabbit lgG Santa mouse Cat# SC2025 IP, IHC https://www.scbt.com/zh/p/normal-mouse-igg?requestFrom=search
CD16/32 (93) BioLegend mouse Cat# 101302 Flow https://www.biolegend.com/en-us/products/purified-anti-mouse-cd16-32-antibody-190
Anti-CD11c (N418) PerCP BioLegend mouse Cat# 117324 Flow https://www.biolegend.com/en-us/products/apc-cyanine7-anti-mouse-cd11c-antibody-3931
Anti-mouse CD11c (N418) APC BioLegend mouse Cat# 117310 Flow https://www.biolegend.com/en-us/products/apc-anti-mouse-cd11c-antibody-1813
Anti-CD11b (M1/70) FITC BioLegend mouse Cat# 101206 Flow https://www.biolegend.com/en-us/products/fitc-anti-mouse-human-cd11b-antibody-347
Anti-CD11b (M1/70) PE BioLegend mouse Cat# 101208 Flow https://www.biolegend.com/en-us/products/pe-anti-mouse-human-cd11b-antibody-349
Anti-CD103 (2E7) BV510 BioLegend mouse Cat# 121423 Flow https://www.biolegend.com/en-us/products/brilliant-violet-510-anti-mouse-cd103-antibody-9063
Anti-H-2Kb (M5/114) BV421 BioLegend mouse Cat# 107632 Flow, IHC https://www.biolegend.com/en-us/products/brilliant-violet-421-anti-mouse-i-a-i-e-antibody-7147
Anti-CD86 (GL-1) FITC BioLegend mouse Cat# 105005 Flow https://www.biolegend.com/en-us/products/fitc-anti-mouse-cd86-antibody-254
Anti-CD8 (53-6.7) PE BioLegend mouse Cat# 100708 Flow https://www.biolegend.com/en-us/products/pe-anti-mouse-cd8a-antibody-155
Anti-CCR1 (S15040E) FITC BioLegend mouse Cat# 152505 Flow https://www.biolegend.com/en-us/products/fitc-anti-mouse-cd191-ccr1-antibody-14455
Anti-CCR2 (SA203G11) BV510 BioLegend mouse Cat# 150617 Flow https://www.biolegend.com/en-us/products/brilliant-violet-510-anti-mouse-cd192-ccr2-antibody-15389
Anti-CCR3 (J073E5) BV421 BioLegend mouse Cat# 144517 Flow https://www.biolegend.com/en-us/products/brilliant-violet-421-anti-mouse-cd193-ccr3-antibody-11896
Anti-F4/80 (BM8) PE BioLegend mouse Cat# 123110 Flow https://www.biolegend.com/en-us/products/pe-anti-mouse-f4-80-antibody-4068
Anti-Ly6G (1A8) APC-Cy7 BioLegend mouse Cat# 127623 Flow https://www.biolegend.com/en-us/products/apc-cyanine7-anti-mouse-ly-6g-antibody-6755
Anti-NK1.1 (PK136) BV421 BioLegend mouse Cat# 108731 Flow https://www.biolegend.com/en-us/products/brilliant-violet-421-anti-mouse-nk-1-1-antibody-7150

Anti-CD3 (145-2C11) APC-Cy7 BioLegend mouse Cat# 100330 Flow https://www.biolegend.com/en-us/products/apc-cyanine7-anti-mouse-cd3epsilon-antibody-6070
Anti-CD4 (GK1.5) APC BioLegend mouse Cat# 100411 Flow https://www.biolegend.com/en-us/products/apc-anti-mouse-cd4-antibody-245
Anti-CD8 (53-6.7) FITC BioLegend mouse Cat# 100706 Flow https://www.biolegend.com/en-us/products/fitc-anti-mouse-cd8a-antibody-153
Anti-IFNg (XMG1.2) PE BioLegend mouse Cat# 505807 Flow https://www.biolegend.com/en-us/products/pe-anti-mouse-ifn-gamma-antibody-997
Anti-IL-4 (11B11) PE BioLegend mouse Cat# 504104 Flow https://www.biolegend.com/en-us/products/pe-anti-mouse-il-4-antibody-893
Anti-IL-17A (TC11-18) FITC BioLegend mouse Cat# 506908 Flow https://www.biolegend.com/en-us/products/fitc-anti-mouse-il-17a-antibody-3534
Anti-PD-1 (RMP1-30) PE BioLegend mouse Cat# 109104 Flow https://www.biolegend.com/en-us/products/pe-anti-mouse-cd279-pd-1-antibody-454
Anti-PD-1 (J43) Bio X cell mouse Cat# BE0033-2 WB https://bxcell.com/product/m-cd279/
Hamster lgG Bio X cell mouse Cat# BE0091 WB https://bxcell.com/product/polyclonal-3/

Eukaryotic cell lines

Policy information about cell lines

Cell line source(s)	HEK293T and 3T3 cell lines were from American Type Culture Collection
Authentication	These cells were authenticated by STR locua analysis by Chinese Type Culture Collection, Wuhan University.
Mycoplasma contamination	These cells were test for mycoplasma negative.
Commonly misidentified lines (See ICLAC register)	No commonly misidentified cell lines were used in the study.

Animals and other organisms

Policy information about studies involving animals; ARRIVE guidelines recommended for reporting animal research

Laboratory animals	KrasLSL-G12D/+ (#008179), Tp53fl/fl (#008462), Lkb1fl/fl (#014143), and Ccl7-/- (#017638) mice were purchased from the Jackson Laboratory. KrasLSL-G12D/+, Tp53fl/fl, and Ccl7-/- mice were crossed to obtain KrasLSL-G12D/+Tp53fl/fl (KP) and KrasLSL-G12D/+Tp53fl/flCcl7-/- (KP7) mice for maintenance and experiments. KrasLSL-G12D/+ and Lkb1fl/fl were crossed to obtain KrasLSL-G12D/+Lkb1fl/fl (KL) mice. The Ccl7IRES-ZsGreen mice were generated by GemPharmatech Co., Ltd through CRISPR/Cas9-mediated genome editing. In brief, the vector encoding guide RNA (GGCACATTTCTTCAAGGCTT) was obtained by in vitro transcription and purification. The gRNAs were incubated with purified Cas9 protein and injected the fertilized eggs (one-cell stage) together with the targeting vector containing the IRES-ZsGreen cassette. The injected fertilized eggs were cultured to the two-cell stage followed by transplantation into pseudopregnant mice. The targeted genomes of F0 mice were amplified by PCR and sequenced. The genomic DNA from PCR positive F0 mice was subject to Southern blot analysis to confirm correct recombination and exclude random insertions of the targeting vector. The correct F0 mice were crossed with wild-type C57BL/6 mice to obtain the F1 Ccl7IRES-ZsGreen mice that were crossed with the KP mice to obtain KP7IRES-ZsGreen mice. The genotyping primers for the Ccl7IRES-ZsGreen allele were listed in Supplementary Table 5. Eight-week-old male and female KP, KP7 or KL mice were intranasally injected with Ad-Cre followed by various experiments. B6.SJL (#002014) mice (8 weeks old) were from the Jackson Laboratory and kindly provide by Dr. Haojian Zhang (Wuhan University). CD11c-DTR mice (#004509) (8 weeks old) were from the Jackson Laboratory and kindly provided by Drs. Xin-Yuan Zhou and Ying Wan (Third Military Medical University). Zbtb46-DTR mice (#019506) (8 weeks old) were from the Jackson Laboratory and kindly provided by Drs. Cliff Yang (Sun Yat-sen University) and Xiao Shen (Zhejiang University). C57B/6 mice (8 weeks old) were purchased from GemPharmatech Co., Ltd (Nanjing, Jiangsu Province). All experimental groups contained male and female mice and all the mice housed in the specific pathogen-free animal facility (12h/12h light and dark cycle, 22oC ± 2oC) at Wuhan University. All animal experiments were performed in accordance with protocols approved by the Institutional Animal Care and Use Committee of Wuhan University.
Wild animals	This study did not involve wild animals.
Field-collected samples	This study did not involve samples collected from the field.
Ethics oversight	All animal experiments were in accordance with protocols approved by the Institutional Animal Care and Use Committee of Wuhan University.

Note that full information on the approval of the study protocol must also be provided in the manuscript.

Human research participants

Policy information about studies involving human research participants

Population characteristics	Four cohorts of human NSCLC samples were collected and analyzed in this study. Cohort 1 contained 18 paired normal and tumor tissues from NSCLC patients who underwent surgery from June to August of 2013 at the Department of Thoracic Surgery, Tongji Hospital. Cohort 2 contained 44 pared tumor and normal tissues from NSCLC patients who underwent surgery from November of 2013 to March of 2014 at the Department of Thoracic Surgery, Tongji Hospital. The tumor and normal tissues (~0.2 g) were washed with PBS, immersed in TRIzol and frozen in liquid nitrogen immediately after surgery. Cohort 3

contained 287 paraffin-embedded tumor tissues collected from January of 2012 through April of 2014 at the Department of Oncology and the Department of Pathology, Tongji Hospital. Patients of cohort 3 were followed up for survival or NSCLC-related death every three months for five successive years. Cohort 4 contained 35 paraffin-embedded tumor tissues that were obtained with CT-guided needle puncture and stained as PD-L1 positive samples. Patients of cohort 4 were diagnosed with advanced NSCLC without EGFR or ALK mutations and progressed after at least one line of chemotherapy. Patients of cohort 4 were subject to pembrolizumab or sintilimab plus platinum-based chemo-reagents treatment and followed up by CT imaging to evaluate the efficacy of treatment. Patients whose tumor sizes were shrunk by more than 30% of the initial sizes were recognized as partial response (PR) to the therapies. Patients whose tumor sizes were enlarged by more than 20% of the initial sizes were recognized as progressive disease (PD) in response to the therapies. Patients whose tumor sizes were neither shrunk nor enlarged than the initial sizes were recognized as stable disease (SD) in response to the therapies. The clinical information of patients from the four Cohorts was included or summarized in Supplementary Tables 1-4 and Supplementary Data. All cases were re-reviewed by pathologists from the Department of Pathology of Tongji Hospital for the confirmation of tumor histology and tumor content.

Recruitment	This study did not involve clinical trials and all patients were told about the research information. Written informed consent was obtained from all patients.
Ethics oversight	This study was approved by the Institutional Research Ethic Committee of Tongji Hospital, Huazhong University of Science and Technology, and the Medical Ethic Committee of the School of Medicine, Wuhan University.

Note that full information on the approval of the study protocol must also be provided in the manuscript.

Flow Cytometry

Plots

Confirm that:

- [x] The axis labels state the marker and fluorochrome used (e.g. CD4-FITC).
- [x] The axis scales are clearly visible. Include numbers along axes only for bottom left plot of group (a 'group' is an analysis of identical markers).
- [x] All plots are contour plots with outliers or pseudocolor plots.
- [x] A numerical value for number of cells or percentage (with statistics) is provided.

Methodology

Sample preparation	Single cell suspensions were incubated with fluorochrome-conjugated antibodies against surface markers in PBS containing 1.5% FBS for 20 min at 4oC and then washed. LIVE/DEAD Fixable Blue Dead Cell Stain Kit from Biolegend was used to exclude dead cells. Cells were then fixed for 30 min at 4oC using Biolegend Cytofix/Cytoperm and washed twice. For intracellular cytokine and Foxp3 staining, cells were stained with fluorochrome-conjugated antibodies in the Transcription Factor staining buffer set (eBioscience, 50-112-9060). The detailed experimental procedures were described in the methods session.
Instrument	BD Fortessa and Celesta
Software	Windows Flowjo 10.6.2 for data analysis; BD FACSDiVa v8.0.1.1 for cell collection
Cell population abundance	We did not sort any population in this study.
Gating strategy	Forward versus side scatter (FSC vs SSC) gating was used to identify cells of interest and exclude debris and dead cells. also, LIVE/DEAD Fixable Dead Cell Stain Kit from Biolegend was used to exclude dead cells. A forward scatter width (FSC-W) vs. forward scatter area (FSC-A) density plot was used to exclude doublets. For cytokine measurement, we used non-stimulated samples as negative controls.

- [x] Tick this box to confirm that a figure exemplifying the gating strategy is provided in the Supplementary Information.
